# Graphene/Reduced Graphene Oxide-Carbon Nanotubes Composite Electrodes: From Capacitive to Battery-Type Behaviour

**DOI:** 10.3390/nano11051240

**Published:** 2021-05-08

**Authors:** Olena Okhay, Alexander Tkach

**Affiliations:** 1 TEMA-Center for Mechanical Technology and Automation, Department of Mechanical Engineering, University of Aveiro, 3810-193 Aveiro, Portugal; 2 CICECO-Aveiro Institute of Materials, Department of Materials and Ceramic Engineering, University of Aveiro, 3810-193 Aveiro, Portugal; atkach@ua.pt

**Keywords:** energy storage, multifunctional materials, composites, graphene, nanostructures

## Abstract

Thanks to the advanced technologies for energy generation such as solar cells and thermo- or piezo-generators the amount of electricity transformed from light, heat or mechanical pressure sources can be significantly enhanced. However, there is still a demand for effective storage devices to conserve electrical energy which addresses the wide range of large stationary applications from electric vehicles to small portable devices. Among the large variety of energy-storage systems available today, electrochemical energy sources and, in particular, supercapacitors (SC), are rather promising in terms of cost, scaling, power management, life cycle and safety. Therefore, this review surveys recent achievements in the development of SC based on composites of such carbon-derived materials as graphene (G) and reduced graphene oxide (rGO) with carbon nanotubes (CNT). Various factors influencing the specific capacitance are discussed, while specific energy and power as well as cycling stability of SC with G/rGO-CNT composite electrode materials are overviewed.

## 1. Introduction

**Figure 1:**
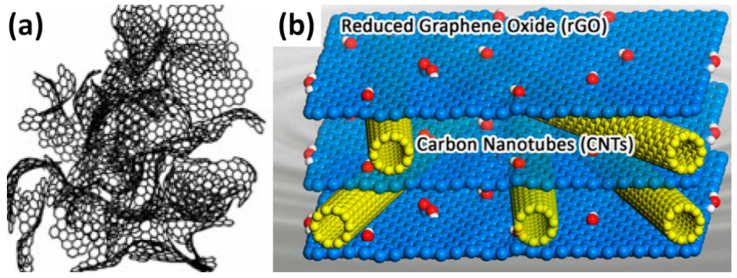
Model of activated carbon based on fullerene-like elements (**a**) (Reproduced with permission of [2]. Copyright Royal Society of Chemistry, 2007) and schematic model of reduced graphene oxide (rGO)/carbon nanotubes (CNT) hybrid structure (**b**) with blue and yellow spheres corresponding to carbon atoms of rGO and CNT, respectively, as well as white and red spheres representing hydroxyl group hydrogen and oxygen atoms, respectively (Reproduced with permission of [10]. Copyright Elsevier, 2015).

Besides the energy transformation, energy storage is one of the most important topics of scientific research today. Similar to the existing commercial batteries, electrochemical capacitors (often called supercapacitors (SC)) are widely studied for their commercial application in electrical cars, portable electronics, etc. The most famous capacitive materials are carbon-based compounds, particularly activated carbon (AC) that is already used for energy storage due to its large surface area and low cost [1]. However, many carbon atoms in AC cannot be accessed by electrolyte ions, thus being wasted in terms of activating their electrochemical functions, due to a very irregular shape of AC, shown in Figure 1a [2]. That decreases the capacitance of the AC electrodes as well as reducing their electrical conductivity (see Table 1).

In addition to AC, a graphene (G), being 2D sp^2^-hybridized carbon sheet, also has a large surface area as shown in Table 1. However, its electrical conductivity is significantly higher, making graphene-related materials very promising for energy storage [3,4,5,6,7,8,9]. However, from G it is easy to form aggregates due to intensive π–π interaction or to restacking forming graphite through van der Waals interactions. If G sheets are stacked together, the electrolyte ions have difficulties gaining access to the inner layers to form electrochemical double layers. Thus, the ions could be accumulated only on the top and bottom surfaces of the sheet agglomerates that can lead to a lower specific capacitance. The problem of graphene sheet agglomerations during the preparation process is valid also for multilayer reduced graphene oxide (rGO) despite lower surface area compared to that of monolayer G. However, the lower price and properties tunable by oxidation degree as well as high electrical conductivity and synergistic effects in composite materials also make rGO useful for electrode development.

As one of the possible ways to prevent the sheets from sticking together is a use of 1D material like carbon nanotubes (CNT) as a spacer (see Figure 1b) [10]. CNT have a readily accessible surface area as well as high conductivity (see Table 1). Since the surface area is known to increase with decrease of the CNT diameter, single-wall carbon nanotubes (SWCNT) are expected to have higher specific capacitance compared to multiwall carbon nanotubes (MWCNT) [5]. However, CNT often stack in bundles and only the outermost portion of CNT can function for ion absorption, whereas the inner carbon atoms are not involved in the process. From another side, pristine CNT with preserved electronic structures can be easily dispersed in graphene oxide (GO) solution without any additives and generate clean, electrically addressable carbon–carbon interfaces. Thus, GO is a “surfactant” to directly disperse CNT, while CNT can prevent the aggregation of graphene sheets as a spacer, and has high conductivity, high surface area, and prospective mechanical properties. Moreover, non-conductive GO without any thermal or chemical reduction was found to be a promising material for SC based on a simulation, which claimed its capacitance decreases with increasing oxidation state [11]. Currently, there are a number of publications on G/rGO-CNT composites with different final parameters as promising electrode materials for energy storage applications, many of which are reviewed and analysed in this work.

## 2. General Information on Energy-Storage Materials

Capacitors and batteries as well as supercapacitors (electrochemical capacitors) can store a charge, possessing, however, different charge storage mechanisms. The dielectric capacitor has electrostatic storage, whereas a battery can be characterized by diffusion mechanism of storage based on reduction-oxidation (redox) processes from used faradaic materials. Moreover, there are clear differences in cyclic voltammograms (CV), providing the current response to a linearly cycled potential sweep, and galvanostatic charge and discharge (GCD) curves, defining how fast a cell is charged or discharged, for these electrodes. Typically, both battery electrodes, anode and cathode, include faradaic materials that result in strong redox peaks in CV, which are clearly visible both for a single electrode and in the full cell (battery) measurements. Moreover, the GCD process of such electrodes as well as devices has long and wide plateaus that can be clearly observed. By contrast with a battery, a dielectric capacitor that stores energy by means of a static charge presents an ideal rectangular shape of CV, the area of which increases with the potential scan rate, and triangular shape of GCD. Moreover, in dielectric capacitors, the current (*i*) flowing through a cell is proportional to the linear variation rate of the voltage (*v*) as *i~v* [12].

**Figure 2:**
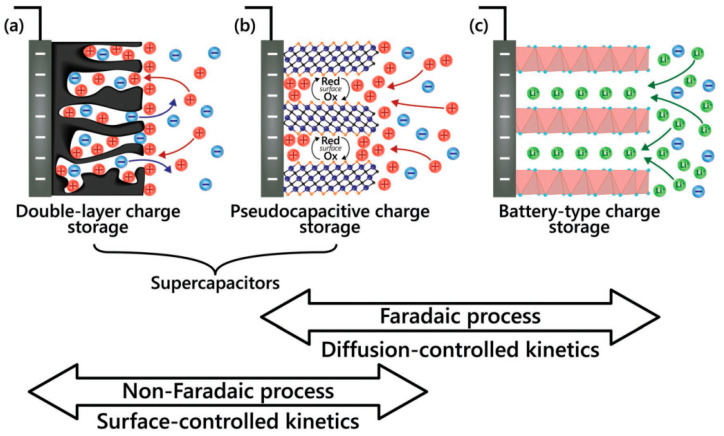
Illustration of the electrode processes occurring at electrical double-layer capacitive (**a**), pseudocapacitive (**b**), and faradaic (**c**) electrodes (Reproduced with permission of [13]. Copyright Wiley, 2019).

**Figure 3:**
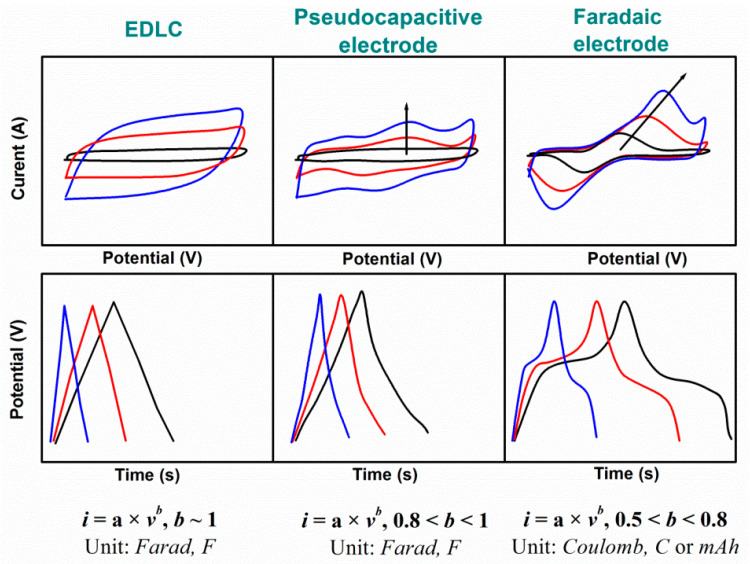
Schematic view of typically reported cyclic voltammograms (CV) and galvanostatic charge and discharge (GCD) curves and applicable information for the G/rGO-CNT electrodes.

At the same time, existing supercapacitors, which can store the energy by electrochemical reactions, include electric double-layer capacitors (EDLC) and pseudocapacitors as can be seen in Figure 2. In EDLC, pure electrostatic charge storage occurs at the electrode–electrolyte interface (see Figure 2a) [13] and their almost rectangular CV increases linearly with the scan rate and has symmetric triangular GCD (see Figure 3, left). In pseudocapacitors, which can involve faradaic materials such as metal oxides or conductive polymers, fast and reversible surface faradaic process such as electron charge-transfer between electrolyte and electrode occurs on/near to the electrode surface (see Figure 2b) that can lead to visible small bulges in CV and small deviations in GCD (see Figure 3, middle). Moreover, pseudocapacitive electrodes show higher capacitance value than EDLC thanks to the faradaic-electron transfer known as reversible surface redox reactions in addition to the non-faradaic charge storage on the surface observed in EDLC. However, the excessive increase of the faradaic contribution (deep intercalation in electrode material (see Figure 2c) can not only increase the capacitance but also dominate diffusion kinetics in the electrodes that is manifested as redox peaks shifted with scan rate in CV patterns, as plateaus in GCD curves (see Figure 3, right), and in longer charge/discharge time [14].

Thus, according to Figure 3, the shape of CV can be rectangular-like for EDLC or can show small redox peaks for pseudocapacitive composites including faradaic materials but with dominant EDLC charge storage. Faradaic capacitance electrodes, being also composites of capacitive materials with faradaic material similar to that of battery electrodes, are characterized by distinct and widely separated peaks in CV with an increase of scan rate. GCD curves of single battery-type electrodes are profoundly non-linear and characterized by plateaus of nearly constant potential corresponding to the potentials, at which the faradaic reduction or oxidation of the metal centres, etc. is occurring in contrast to GCD of pseudocapacitor with slight surface redox on the top of the electrode.

The total current of CV measurements under a potential sweep rate (*i*(*v*)) for composites with faradaic materials consists of two parts [15]. One part is a current related to the double-layer charge at the electrolyte interface or to initial fast faradaic reactions on the exposed electrode surface (*i_cap_*). Another part is the current related to the slow diffusion-controlled process (*i_dif_*). The capacitive contribution and the diffusion-controlled contribution can be calculated following a power-law relationship with the sweep rate (*v*) according to Equation (1):

*i*(*v*) = *i*_*cap*_ + *i*_*dif*_ = *a* × *v^b^*, (1)

where *a* and *b* are variable parameters. *b* values can be estimated from the slope of *log*(*i*) vs. *log*(*v*) plot. The value *b* = 1 corresponds to the presence of the fast surface redox reaction and charge/discharge process inherent to EDLC when diffusion contribution is absent and CV show linear current response dependency on the scan rate (*i~v*) [14]. At the same time, the peak current response of a battery-type electrode with strong redox peaks will be proportional to the square root of the scan rate (*i~v^1/2^*) and in this case *b* = 0.5.

Thus, the number of the electrodes combining capacitive and faradaic materials reveals 0.5 < *b <* 1, exhibiting either strong capacitive or battery-type behaviour. Moreover, as also seen from Figure 3, electrodes with 0.8 < *b* < 1 are considered as pseudocapacitive materials having predominantly capacitive storage [16,17] in contrast to the electrodes with 0.5 < *b* < 0.8 with dominant faradaic (battery-type) behaviour. Thus, to understand the dominant storage mechanism in composite electrode (capacitive or faradaic/battery-type), the parameter *b* needs to be calculated in addition to correct and deep analysis of CV at different scan rates, GCD curves, as well as electrochemical impedance spectra (EIS) including the slope of Nyquist plot, etc.

**Figure 4:**
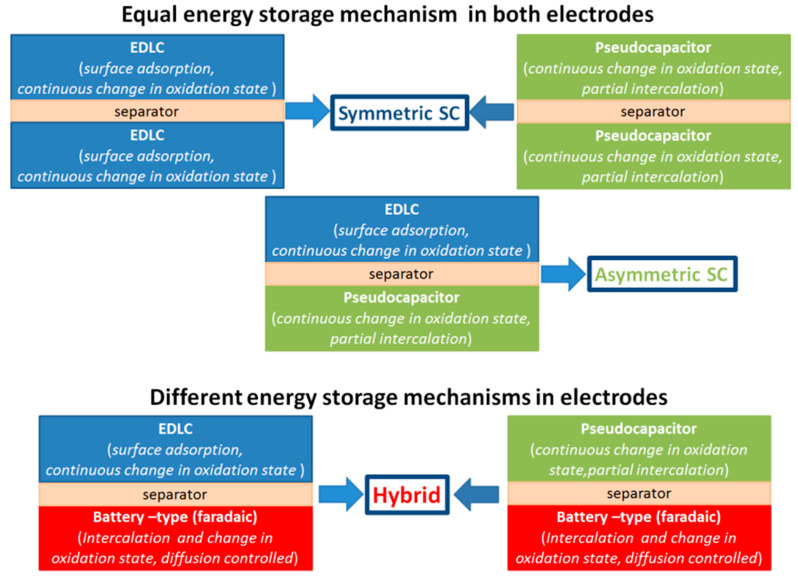
Schematic view of possible combinations of electrodes and final devices fabricated on them.

Moreover, since the energy storage cell consists of two electrodes, different combination of electrodes can give a device with different charge storage mechanism as can be seen in Figure 4. According to Figure 4, the combination of two EDLC electrodes or two pseudocapacitive electrodes results in a symmetric capacitive device. At the same time, an asymmetric design of storage device, consisting of the EDLC electrode together with the pseudocapacitive one, can gain from the enlarged voltage window covering the windows of two different electrodes, still keeping the capacitive mechanism of charge storage as a dominant one [18]. However, in the case of combining two electrodes with different storage mechanism (EDLC and battery-type or pseudocapacitor and battery-type) this mechanism cannot be kept and the final hybrid device will store energy in a different way to capacitive materials. Based on that, energy and power densities of hybrid devices must be compared directly with that reported for batteries, since their electrochemical performance must be higher in advance compared to that of symmetric and asymmetric supercapacitors because of faradaic materials, which are dominant in the battery-type electrodes. Thus, the charge storage mechanism of single electrode needs to be clarified before fabrication of the energy storage device. The best way to understand it is the detailed analysis of their characteristics, particularly CV, *b* parameter, GCD, Nyquist plot.

## 3. Composite Capacitive Electrodes Based on Graphene/Reduced Graphene Oxide-Carbon Nanotubes (G/rGO-CNT)

Both G/rGO and CNT are capacitive materials and their composite electrodes also present EDLC type of energy storage with specific capacitance values varying up to 375 F/g [19] as shown in Table S1 in Supplementary Materials. Typically, rGO-CNT-based electrodes show not an ideal but almost a rectangular shape of CV with slight deviation due to an electrode resistance contribution, including ion transfer resistance or electrode material resistance, or even some surface redox associated with oxygen functional groups especially at high scan rate.

Enhancement of the electrochemical performance G/rGO-CNT electrodes and, respectively, a final device, can be undertaken not only by addition of external components, but also by modification/functionalization of G/rGO and/or CNT. Thus, the properties of graphene-based materials are known to depend on the synthesis method and reduction process. In particular, for the preparation of mixed G/rGO-CNT-based composites, carbon nanotubes were combined with:

- commercially available graphene or graphene grown previously or directly in the process;
- rGO obtained after reduction of GO by high temperature, or by hydrazine hydrate (H_6_N_2_O), ammonium solution (NH_4_OH), vitamin C, acids, etc.

Moreover, rGO-CNT-based electrodes were fabricated both on substrates, such as glassy carbon electrode (GCE), Ni foams, metal foils, stainless steel; carbon-based supports as carbon cloth/carbon paper, or graphite substrates; polyvinylidene fluoride (PVDF)-treated paper; Si; plastic; indium tin oxide, etc. and without support as freestanding, i.e., by vacuum filtration, frying or as fibres by injections, etc. Besides such important parameters as the annealing temperature or chemicals used to control the oxygen functional groups content, a ratio between G/rGO and CNT as well as their typology and electrolyte type are also very important. Their influence will be overviewed in the subchapters below. In addition, most of the studies involved not only G/rGO and CNT but also some conductive additives and polymer binders in the similar to commercial battery fabrication process (typically, in the 80:10:10 weight ratio between active material (AM), conductive carbon-family-material and polymer).

### 3.1. Temperature Effect on G/rGO-CNT-Based Composite Electrodes

**Figure 5:**
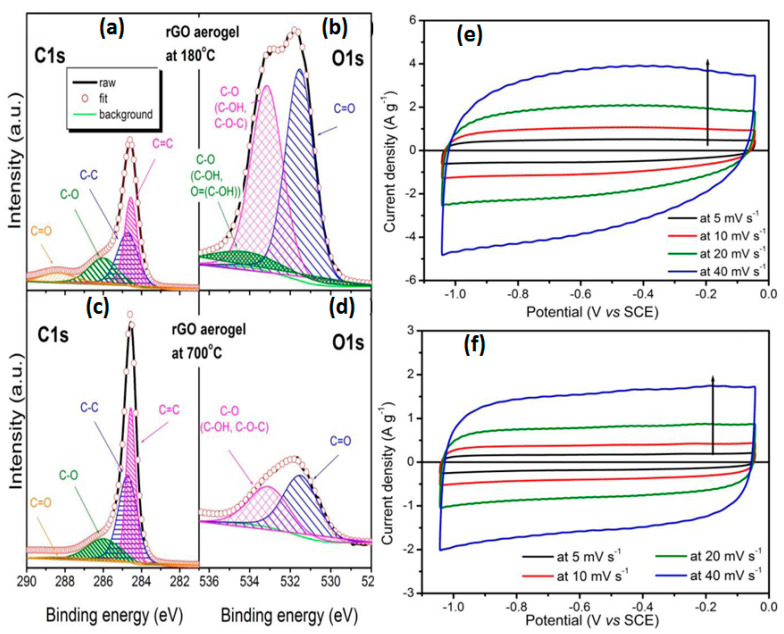
Carbon (C1s) (**a**,**c**) and oxygen (O1s) (**b**,**d**) spectra obtained by X-ray photoelectron spectroscopy (XPS) on graphene oxide (GO) aerogel reduced at 180 °C in vacuum (**a**,**b**) and at 700 °C in Ar (**c**,**d**). Cyclic voltammetry profiles at different scan rates for the rGO-CNT-based composite electrodes on carbon cloth with 180 °C (**e**) and at 700 °C (**f**) rGO (Reproduced with permission of [20]. Copyright Elsevier, 2020).

The freeze-dried GO aerogels reduced at 180 and at 700 °C were used together with SWCNT for the fabrication and comparative study of rGO-CNT composite electrodes with PVDF binder on carbon cloth by Okhay et al. [20]. X-ray photoelectron spectroscopy (XPS) data of the rGO aerogels shown in Figure 5a present a strongly reduced oxygen functional group after heat treatment at 700 °C that is supported by the almost ideal rectangular CV curves for the high temperature-reduced aerogel (see Figure 5b) by contrast with that of rGO aerogel annealed at 180 °C that still show a bulge of surface redox on EDLC (see Figure 5c). Moreover, the specific capacitance of 129 F/g reported for the electrodes made of 180 °C rGO is much higher than that of 41 F/g at 0.1 A/g for 700 °C rGO [20].

**Figure 6:**
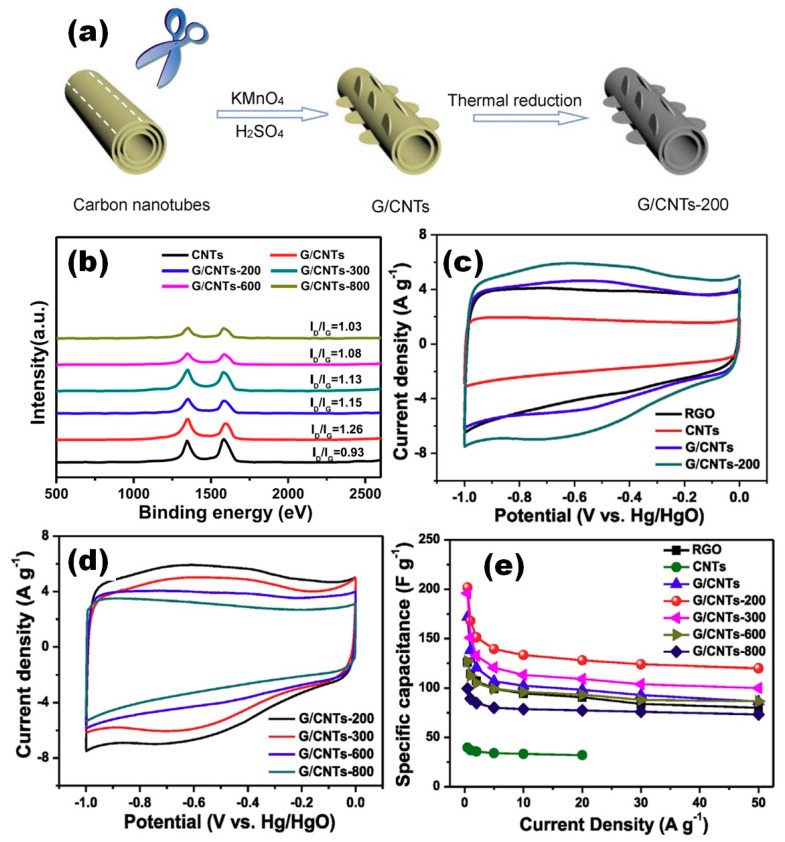
Schematic illustration of the synthetic route of reduced graphene oxide with carbon nanotubes (mentioned here as G/CNTs) after annealing at 200 °C (G/CNTs-200) (**a**). Raman spectra of studied electrodes (**b**). CV curves measured at 50 mV/s on rGO, CNT, G/CNT and G/CNTs-200 (**c**), as well as G/CNT annealed at 300, 600 and 800 °C (G/CNTs-300, G/CNTs-600, and G/CNTs-800, respectively) (**d**). Specific capacitance of rGO, CNTs, G/CNTs, G/CNTs-200, G/CNTs-300, G/CNTs-600 and G/CNTs-800 as a function of current density (**e**) (Reproduced with permission of [21]. Copyright Elsevier, 2018).

A systematic study of temperature influence on functionalized graphene nanosheets/carbon nanotubes networks (G/CNT) synthesized through chemical oxidation of CNT followed by thermal reduction according to schematic illustration in Figure 6a was reported by Ding et al. [21]. The external walls of CNT in the G/CNT structure annealed at 200 °C were observed as unzipped and transformed into functionalized graphene nanosheets, while the inner walls were not unzipped and kept the tubular structure. Freeze-dried G/CNT mixture was heat treated at 200, 300, 600 and 800 °C for 2 h under N_2_ flow and mixed with carbon black (CB) and poly (tetrafluoroethylene) (PTFE) before the covering on Ni foam.

Based on Raman spectra, shown in Figure 6b, the I_D_/I_G_ ratio was found to increase after chemical oxidation from 0.93 to 1.26, revealing that a large number of oxygen functional groups were introduced during the unzipping process. However, after thermal reduction at 200 °C partially unstable oxygen functional groups were removed as can be also seen in Figure 6b, leading to an increase of the integrated area of CV in comparison with as-prepared material (see Figure 6c). The fact that after thermal reduction of G/CNT at 200 °C conjugated carbon networks were restored was also mentioned. However, after thermal reduction at T > 200 °C (see Figure 6b) the integrated area of CV was also suppressed (see Figure 6d) despite the fact that unstable oxygen functional groups were progressively reduced. Therefore, the highest specific capacitance of 202 F/g at 0.5 A/g was reported for electrodes with G/CNT after heat treatment at 200 °C (see Figure 6e). Moreover, CV curves of G/CNTs and G/CNTs-200 electrodes show obvious redox humps in Figure 6c, meaning that the capacitances come both from EDLC and pseudocapacitance due to the reversible redox reactions among the surface oxygen functional groups [21].

### 3.2. Effect of CNT Length

**Figure 7:**
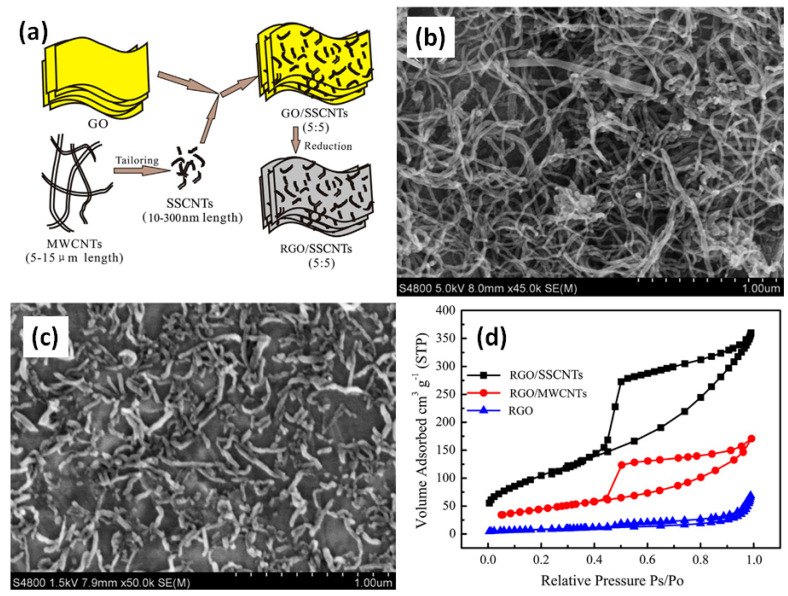
Schematic illustration of the formation steps of the multilayer rGO/super short CNT (SSCNT) architecture (**a**). Scanning electron microscope (SEM) images of raw multiwall CNT (MWCNT) (**b**) and SSCNT (**c**). Nitrogen sorption isotherms obtained at 77 K (**d**) (Reproduced with permission of [22]. Copyright Elsevier, 2013).

The size of CNT can change the value of specific capacitance at least 3 times as was reported by Zeng et al. [22]. To increase the utilization of closed pore volumes of CNT and prevent the stacking of rGO, multiwall CNT (MWCNT) are tailored into super short CNT (SSCNT) with aspect ratio of less than 5 by an ultrasonic oxidation-cut method (see Figure 7a). After mixing SSCNT with rGO and PVDF, it was coated onto titanium plate. The morphologies of the composite with MWCNTs and SSCNT were found very different by scanning electron microscopy (SEM) as shown in Figure 7b,c. The length reduction from 5–15 μm to 10–300 nm led to rich structural features, such as nanoscale length, open ends, abundant carbon atoms on the edge, quasi-0D characteristic and so on [22]. Moreover, the introduction of SSCNT increased the specific surface area of rGO to 370 m^2^/g that was higher than 171 m^2^/g after addition of MWCNT (see Figure 7d). Thus, shorter CNT can be more uniformly distributed on the surface of rGO and form 3D multilayer architecture that leads to an increase of specific capacitance from 88 F/g to 251 F/g at 50 mV/s [22].

### 3.3. CNT Concentration in G/rGO-CNT-Based Composite Electrodes

**Figure 8:**
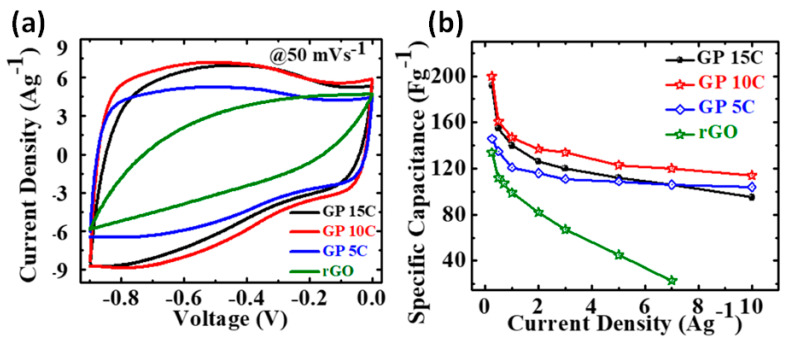
CV curves at the scan rate of 50 mV/s (**a**) and specific capacitance as a function of current density determined from GCD curves (**b**) for rGO and rGO-CNT composites with 5, 10 and 15 wt.% of CNT electrodes (designated as GP5C, GP10C, and GP15C) in KOH electrolyte [24].

Based on results reported by Lu et al. 16 wt.% CNT was the optimal concentration in the range 0 ÷ 50 wt.% for rGO-CNT freestanding electrodes to obtain the highest capacitance of 265 F/g at 0.1 A/g [23]. A lower content of 10 wt.% CNT was chosen by Kumar et al. for dried composite pressed into Ni foam as the optimal concentration of CNT to obtain the rectangular-like CV (see Figure 8a) and the highest specific capacitance (see Figure 8b) [24]. Among the studied CNT concentrations from 0 to 66.7 wt.%, 12.5 wt.% CNT was used to get the highest capacitance for rGO-CNT obtained by vacuum filtration (VF) and pressed into Ni foam [25]. Specific capacitance of 132 F/g was reported by Lee et al. for rGO-CNT with 11 wt.% CNT prepared on glassy carbon electrode among other composites with CNT concentration varied from 6 to 50 wt.% [26]. Thus, the optimal amount of CNT for rGO-CNT mixed composites was found to be from 10 to 16 wt.%. However, in the case of preparation of a CNT layered structure onto graphite paper by electrophoretic deposition, significantly higher content of 40 wt.% CNT in suspension was used to obtain the highest capacitance in that work of 87 F/g at 5 mV/s [27].

### 3.4. Influence of Electrolyte Type and Potential Window

**Figure 9:**
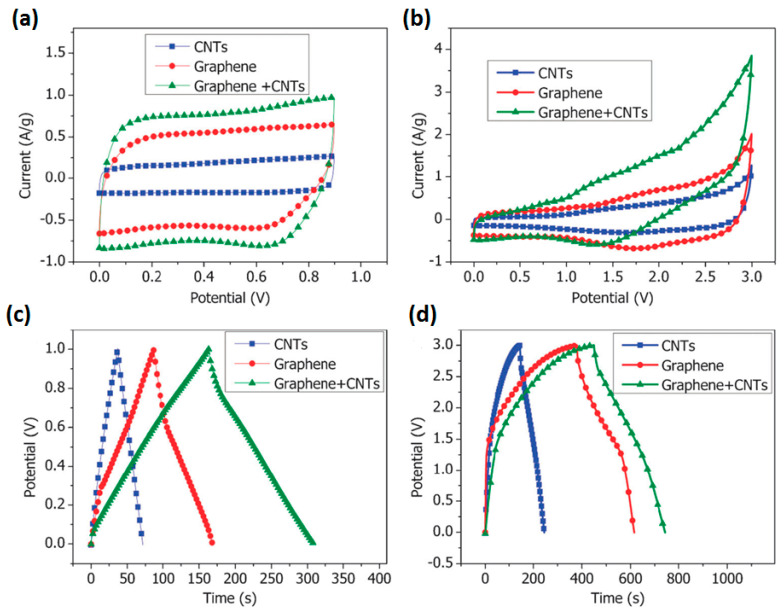
Electrochemical properties of various electrodes made of CNTs, graphene, and graphene/CNT composite. (**a**) Cyclic voltammetry curves in the aqueous KCl electrolyte at a scan rate of 10 mV/s. (**b**) Cyclic voltammetry curves in 1 M TEABF_4_/PC electrolyte at the same scan rate of 10 mV/s. (**c**) Galvanostatic charge/discharge curves in the aqueous electrolyte at a charging current of 500 mA/g. (**d**) Galvanostatic charge/discharge curves in the organic electrolyte at the same charging current of 500 mA/g (Reproduced with permission of [29]. Copyright Royal Society of Chemistry, 2011).

Based on the published research data, G/rGO-CNT-based electrodes were studied in both aqueous and non-aqueous electrolytes as well as in liquid and solid states. Aqueous electrolytes are less expensive, not flammable, and not/less toxic in contrast to e.g., organic electrolytes used in commercially available batteries/SC [28]. Many researchers used aqueous liquid and solid electrolytes: acids such as H_2_SO_4_ or H_3_PO_4_ or alkaline KOH or neutral Na_2_SO_4_, KCl, LiClO_4_, Li_2_SO_4_. Non-aqueous electrolytes were reported in the several publications as organic electrolytes TEABF_4_ (tetraethylammonium tetrafluoroborate) and Et_4_NBF_4_-AN (tetraethylammonium tetrafluoroborate—acetonitrile), and as ionic liquids EMIM-BF_4_ (1-ethyl-3-methylimidazolium tetrafluoroborate) and EMI-TFSI (1-ethyl-3-methylimidazolium bis(trifluoromethylsulfonyl)imide). However, the results obtained, particularly CV form and size, indicate different behaviour of G/rGO-CNT in different electrolytes. Cheng et al. studied supercapacitor fabricated on freestanding rGO-CNT in neutral electrolyte KCl, organic electrolyte TEABF_4_ in propylene carbonate (PC) and ionic liquid EMI-TFSI [29]. CV curve in the organic electrolyte TEABF_4_/PC does not exhibit rectangular geometry due to the larger resistance in the organic electrolyte, redox groups such as hydroxide group and carboxyl (see Figure 9b) by contrast with CV in KCl (see Figure 9a) and in EMI-TFSI. This corresponds to GCD curves that have shown irregular shape in organic electrolyte (see Figure 9d) but triangular form in KCl (see Figure 9c) [29].

**Figure 10:**
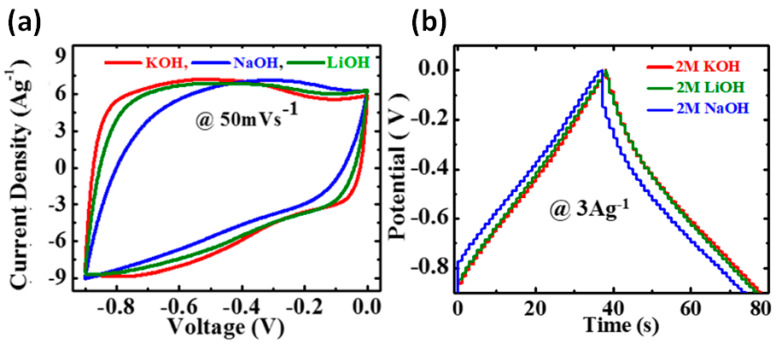
CV curves at 50 mV/s (**a**) and GCD curves at 3 A/g (**b**) of rGO-CNT film with 10 wt.% of CNT (GP10C) in aqueous KOH, LiOH, and NaOH electrolytes [24].

A near rectangular shape of CV and slight asymmetry in GCD curves was observed by Kumar et al. for filtered out rGO-CNT pressed into Ni foam and studied in such electrolytes as KOH, NaOH and LiOH (see Figure 10) [24]. Small deviation from the ideal rectangular CV and triangular GCD curves was explained by the occurrence of some faradaic reaction at the surface ascribed to the oxygen-containing functional groups attached to rGO sheets and functionalized CNT. As shown in Figure 10a, the highest integral area and more rectangular CV curves were observed during the test of electrodes in KOH that can be associated with a smaller hydrated ionic radius and higher ionic conductivity of K^+^ ion in comparison to that of Na^+^ and Li^+^ ions [24]. On the other hand, the ionic mobility enhanced by a lower hydrated ionic radius of K^+^ ion gains access to the electrode surface, resulting in an improved electrochemical performance of rGO-CNT electrode [24].

**Figure 11:**
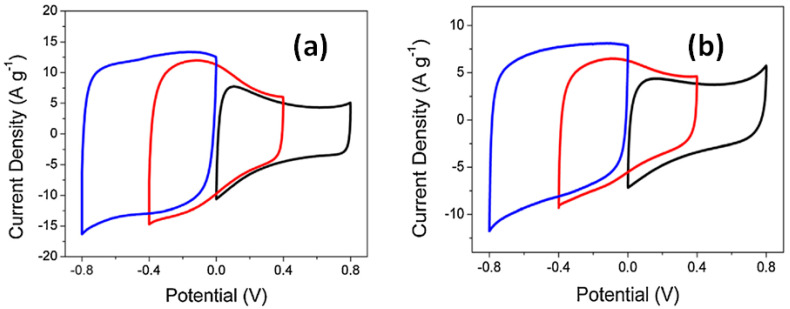
CV curves of rGO-CNT electrode with ratio 10:1 (designated as GC01) obtained in different potential windows at 50 mV/s in Na_2_SO_4_ electrolyte (**a**) and NaCl electrolyte (**b**) (Reproduced with permission of [10]. Copyright Elsevier, 2015).

Cui et al. reported that the CV shape of rGO-CNT composite studied in a positive potential window is different from that tested in a negative potential window (see Figure 11). CV curves of rGO-CNT coated on Ti foil with irregular shape observed from 0 to +0.8 V as well as from −0.4 V to +0.4 V both in Na_2_SO_4_ (see Figure 11a) and NaCl electrolyte (see Figure 11b) indicated the existence of EDLC and pseudocapacitance in opposite to almost rectangular CV in potential window from 0 to −0.8 V [10]. Moreover, integrated areas of CV in Na_2_SO_4_ were obviously higher than that in NaCl. Correspondingly, the specific capacitance was also different and the highest value was obtained for the composite tested in a negative potential window from 0 to −0.8 V in Na_2_SO_4_. Thus, according to Cui et al. the rGO-CNT electrode has shown great potential to be used as a negative electrode for energy-storage devices [10].

## 4. Modified G/rGO-CNT Electrodes with Faradaic Contribution

### 4.1. Nitrogen Doping

One of the popular directions today is modification of G/rGO-CNT by nitrogen (N) due to its atomic size and strong valence bonds, which are similar to those characteristics of carbon atoms [30,31]. Pyrolysis of GO with a low-cost *N* source is a versatile method for large-scale production of *N*-doped graphene with flexible control over the *N*-bonding configurations. *N*-doped G/rGO-CNT structures on different substrates were reported as high-performance supercapacitor electrode materials. Different nitrogen-containing materials such as polyacrylonitrile, acetonitrile, melamine, etc. are commonly used as the nitrogen precursor.

**Figure 12:**
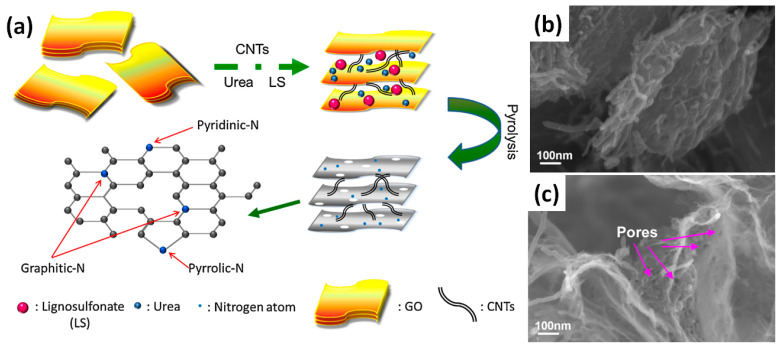
Schematic representation of preparation procedure of porous N-doped rGO-CNT (PNGC) composite (**a**). SEM images of lignosulfonate (LS) modified rGO-CNT (**b**) and of that after the addition of urea and heat treatment at 800 °C (**c**) (Reproduced with permission of [32]. Copyright Elsevier, 2015).

Significant enhancement of the specific capacitance of rGO-CNT composites on GCE after N-doping was reported by Lin et al., when initial value of 10 F/g obtained for rGO-CNT has grown to 168 F/g at 0.5 A/g for N-doped rGO-CNT (designated as NGC) after addition of urea and low-cost lignosulfonate (LS) (see Figure 12) [32]. Adding only LS to the mixture of GO and CNTs (designated as LGC), the obtained graphene sheets in LGC composite were very thick (see Figure 12b).

The result may be attributed to the three-dimensional structure of macromolecular LS. However, after the addition of urea into the mixture of N-doped GO and CNTs, the graphene sheets in NGC were found to be thinner and looser. Moreover, more porous N-doped rGO-CNT (PNGC) obtained after addition of both LS and urea with further heat treatment at 800 °C have shown CV with the highest integrated area that corresponds to the highest capacitance of 246 F/g at 0.5 A/g (see Table S2 in Supplementary Materials for details). During the annealing process, excess urea molecules decompose abundant gases, which open the space between graphene sheets and prevent graphene sheets from stacking tightly. Furthermore, after the addition of LS and urea into the mixture of GO and CNTs, many pores on the surface of thin graphene sheets in PNGC are observed, as shown by arrows in Figure 12c. In addition, the CV curve of rGO-CNT shows a pair of redox peaks, which may be attributed to the residual carboxyl and hydroxyl groups of CNT via oxidation process. CV curves of NGC and PNGC can also be seen to exhibit nearly rectangular shapes and have some peaks as well, ascribed to the combination of electrical double-layer capacitance and faradic pseudocapacitance from nitrogen doping and residual carboxyl and hydroxyl groups [32]. Close capacitance value of 176 F/g at 0.5 A/g was reported for composite electrodes on Ni foam made of N-doped rGO-CNT by addition of polydopamide (PDA), acetylene black (AB) and PVDF [33].

### 4.2. Addition of Conductive Polymers

#### 4.2.1. G/rGO-CNT with Polypyrrole

**Figure 13:**
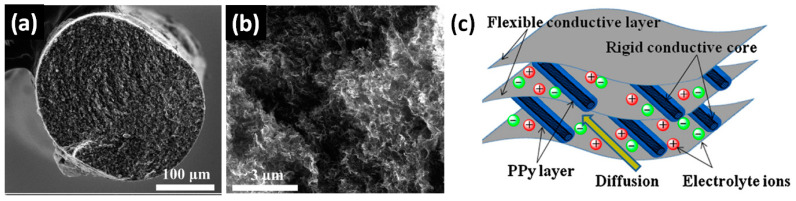
Cross-sectional SEM image of the rGO/MWCNT fibre at low (**a**) and high (**b**) magnifications (Reproduced with permission of [34]. Copyright American Chemical Society, 2017). Schematic representation of the microstructure and energy storage characteristics of the rGO-CNT-polypyrrole (PPy) film (**c**) (Reproduced with permission of [35]. Copyright Elsevier, 2011).

Polypyrrole (PPy) has been extensively studied by many research groups due to its particular advantages with regard to low cost, environmental friendliness, high capacitive capability and easy processing. Typically G/rGO-CNT-PPy composite electrodes were fabricated by the in situ polymerization method. Pseudocapacitive composites of rGO-CNT with PPy were obtained by Wang et al. as fibre electrode [34] and by Lu et al. as freestanding electrode [35] as well as a composite with PTFE onto graphite substrate [36]. In the work of Wang et al., GO-CNT fibres (see Figure 13a) reduced by vitamin C at 90 °C have shown specific capacitance of 10.8 F/cm^3^ at 0.01 V/s in LiCl electrolyte that was increased up to 25.9 F/cm^3^ after covering by PPy [34] (see Table S3 in Supplementary Materials for details).

At the same time Lu et al. measured composite made of rGO, poly(sodium 4-sterene sulfonate) (PSS) functionalized CNT and PPy prepared as freestanding electrode [35] and as electrode on graphite substrate mixed with CB and PTFE (see Figure 13b) [36]. PSS containing a hydrophilic group (–SO_3_) was demonstrated to be strongly and uniformly adsorbed on the surface of rGO-CNT during the modified process that leads to high stability and dispersion of the functionalized rGO-CNT within the aqueous solution. Simultaneously, the sulfonic groups with negative charges extending in the solution provide a number of coordinating sites onto rGO-CNT surface. Such coordinating sites can be used to effectively tether and absorb more monomer PPy and facilitate the following “homogeneous” deposition of PPy particles on the electrode surface. Corresponding rGO-PSS-CNT-based composite electrodes presented capacitive behaviour with specific values of 211 F/g and 361 F/g at 0.2 A/g for freestanding and graphite substrate supported electrode, respectively (see Table S3 in Supplementary Materials for details). That could be explained by an increasing amount of PPy from ~40 wt.% for the freestanding electrode to more than 70 wt.% that on graphite substrate. In the case of further PPy concentration increase the specific capacitance can continue to grow to 453 F/g at 5 mV/s as reported by Aphale et al. for rGO-CNT-PPy electrode using more than 99 wt.% PPy [37].

#### 4.2.2. G/rGO-CNT with Polyaniline

**Figure 14:**
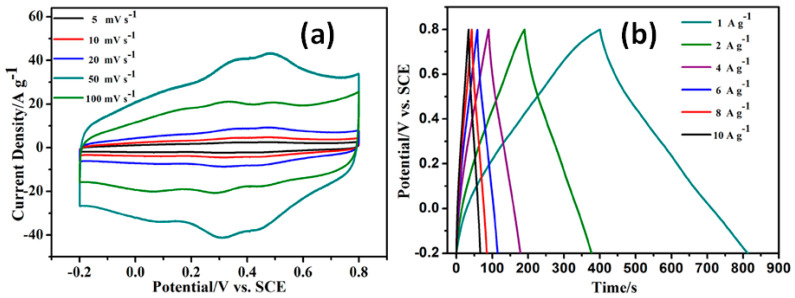
CV curves at different scan rates (**a**) and GCD curves at different current densities (**b**) of rGO/CNT/PANI (Reproduced with permission of [38]. Copyright Elsevier, 2018).

Polyaniline (PANI) is a main conductive polymer with high environmental stability, redox reversibility, electroactivity and unusual doping/de-doping chemistry. PANI as a component of rGO-CNT composite can initiate the pseudocapacitance from the faradaic contribution of its redox nature that, together with EDLC of rGO-CNT, leads to the electrode capacitance enhancement. Typically, PANI can be obtained by an in situ polymerization process using the dissolved aniline monomer [38,39,40,41,42,43]. By this method the total surface of freestanding rGO-CNT paper [43] and fibre electrode [42] was covered by PANI that resulted in the capacitance of 138 F/g at 0.2 A/g and 193 F/cm^3^ at 1 A/cm^3^, respectively (see Table S4 in Supplementary Materials for details). Higher specific capacitance of 359 F/g at 1 A/g was obtained by Huang et al. for electrodes prepared by mixing hydrazine-reduced GO, CNT and aniline to obtain a composite with 80 wt.% PANI [38] and with larger intercalation compared with PANI coating just mentioned above. Then slight surface redox including response from PANI can be observed in CV curves but the corresponding peaks are symmetrical and do not shift with the increasing scan rate (see Figure 14a). That fact together with symmetric and triangular CGD curves (see Figure 14b) supported the dominant capacitive behaviour in these electrodes [38]. In addition, according to our estimation of the *b* parameter for these electrodes it was found to be ~0.9 that is close to *b* = 1 associated with capacitor behaviour.

**Figure 15:**
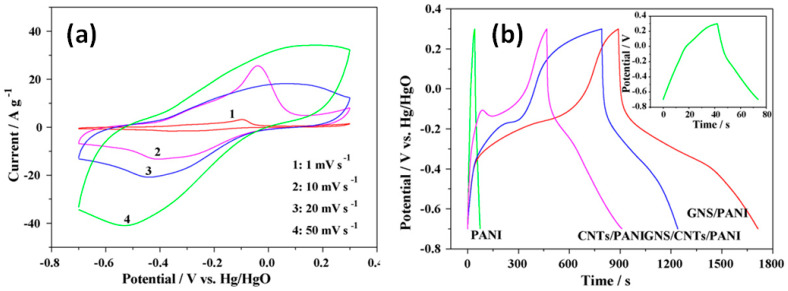
CV curves of rGO-CNT-PANI (GNS/CNTs/PANI) composite at scan rates of 1, 10, 20 and 50 mV/s (**a**) and GCD curves of pure PANI and its composites with CNT (CNTs/PANI), rGO (GNS/PANI) and both of them (GNS/CNTs/PANI) at 2 mA/cm^2^ (**b**) (Reproduced with permission of [41]. Copyright Elsevier, 2009).

At the same time, similar mixture of hydrazine reduced GO (marked as GNS in Figure 15), CNT and aniline, to obtain final composite including also CB and PTFE but with fraction of PANI lowered to ~64 wt.%, was found to present significantly higher capacitance of 1035 F/g at 1 mV/s [41]. However, the appearance of strong redox peaks in CV, shifted with the increasing scan rate (see Figure 15a), and far from symmetrical triangular GCD curve shapes, approaching that with plateau (see Figure 15b), are expected not for materials with capacitive storage mechanism but rather for faradaic materials [41].

Moreover, also high specific capacitance of 987 F/g at 0.5 A/g was reported by Tran et al. for rGO-CNT-PANI prepared by the hydrothermal (HT) method at 180 °C and mixed with mesoporous carbon (MC) and Nafion (with PANI content lowered to ~26 wt.% in final composite) before covering onto carbon paper [39], while value of 638 F/g at 0.5 A/g was measured by Liu et al. for freestanding electrodes fabricated by mixing and filtration of CNT with graphene nanosheets already covered by PANI in an autoclave at 250 °C to form nanorods (with >50 wt.% PANI in composite) [40]. However, these rGO-CNT-PANI composites with high specific capacitance have shown *b* value much lower than 1, being thus not associated with capacitive behaviour in pseudocapacitive materials. In addition, the slope of the EIS Nyquist plot presented by Tran et al. for rGO-CNT-PANI-MC-Nafion electrode on carbon paper was closer to 45° than to 90° [39] which means a strong faradaic contribution in the analysed electrodes. Thus, rGO-CNT-PANI electrodes reported by Liu et al. [40], Tran et al. [39], and Yan et al. [41] (see Table S4 in Supplementary Materials for details) have shown a dominant diffusion-controlled mechanism of energy storage that explains the obtained high value of specific capacitance.

### 4.3. Influence of Metal Catalysts, Metal Oxides and Hydroxides

#### 4.3.1. G/CNT Grown with Me-Catalysts

**Figure 16:**
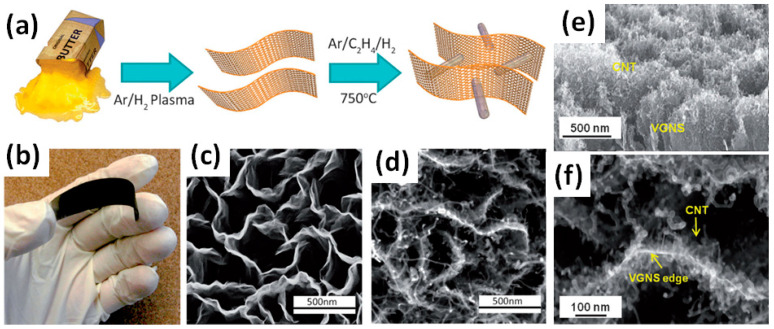
Schematic for the direct growth of CNT onto vertical graphene nanosheets (VGNS) (**a**). Photograph of the as-grown VGNS/CNTs on a flexible graphite substrate (**b**). SEM micrograph of pristine VGNS prior to CNT growth (**c**). SEM micrograph of the final hybrid VGNS/CNTs nanoarchitecture in which the graphene nanosheets were decorated with a high density of CNT (**d**). Cross-sectional (**e**) and high-resolution (**f**) SEM images of the VGNS/CNT (Reproduced with permission of [44]. Copyright Wiley, 2014).

Co, Mo, Al/Fe_2_O_3_, Au and other metals were reported as catalysts used for G or CNT growth for G/CNT electrodes [44,45,46]. Seo et al. formed vertical graphene nanosheets (VGNS) by the plasma transformation of a commercially available natural precursor butter as illustrated in Figure 16a [44]. The plasma was essential in the process to break down the carbon-containing molecules in butter and reconstruct them into ordered and vertical graphitic structures (see Figure 16c). The growth of CNT was then performed in a thermal chemical vapour deposition (CVD) process after the deposition of a Co/Mo catalyst on VGNS. The as-grown VGNS/CNTs hybrid structure on a flexible graphite substrate is presented in Figure 16b. The SEM and transmission electron microscopy (TEM) images of pure VGNS and the VGNS-CNTs obtained after the direct growth process are shown in Figure 16d,f. An inherently open, 3D network with dense and uniform graphene nanosheets was clearly observed to cover the entire surface of the graphite paper [44]. Measured VGNS-CNTs electrodes have shown a specific capacitance of 278 F/g at 10 mV/s (see Table S5 in Supplementary Materials for details) and CV curves with typical shape for EDLC material without redox peaks from Co and Mo used as catalysts. Moreover, neither Co nor Mo was detected by XPS analysis of this structure. GCD curves and Nyquist plot also indicated capacitive energy storage mechanism in the current electrode with the negligible electrochemical contributions of Co and Mo nanoparticles [44].

**Figure 17:**
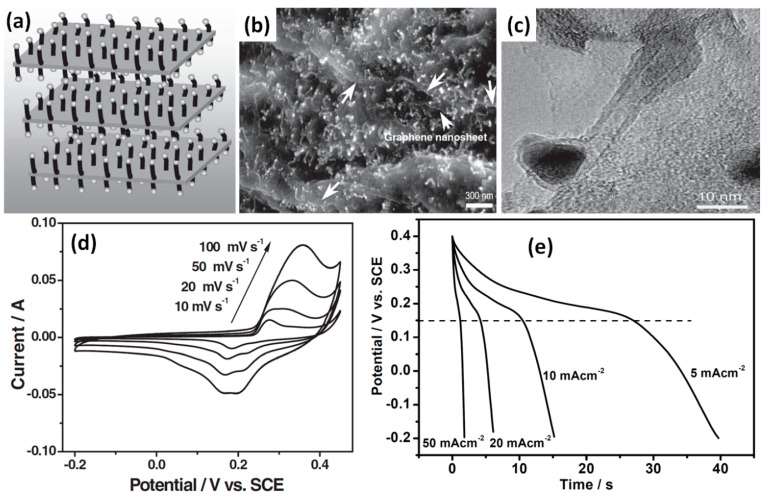
Illustration of the formation of hybrid materials with CNT grown in between graphene nanosheets, showing stacked layers of graphene oxide with Co catalyst particles adhered onto the layer surface after deposition (white points) and CNT in between graphene layers after growth (black tubes) (**a**). SEM images (**b**) and transmission electron microscopy (TEM) image of Co-rGO-CNT (**c**). CV results measured at scan rates of 10, 20, 50, and 100 mV/s (**d**). GCD curves of composite electrode at various current densities (5–50 mA/cm^2^) in KOH solution (**e**) (Reproduced with permission of [45]. Copyright Wiley, 2010).

At the same time, Fan et al. mixed GO with Co(NO_3_)_2_ before growing vertical CNT by CVD at 750 °C with Fe/Al_2_O_3_ as catalyst (see Figure 17a) [45]. In this case the sandwich structure was reported with vertical CNT grown between graphene sheets as can be seen in Figure 17b,c. In opposite to work by Seo et al. where Co/Mo catalyst were not detected by XPS or in an electrochemical study [44], Fan et al. have shown visible Co-based catalysts resided at the top of CNT (see Figure 17e,f). Moreover, strong redox peaks in CV curves shown in Figure 17d as well as nascent plateau in GCD shown in Figure 17e were visible suggesting the high pseudocapacitance of cobalt hydroxide that resulted in measured specific capacitance of 385 F/g at 10 mV/s [45].

**Figure 18:**
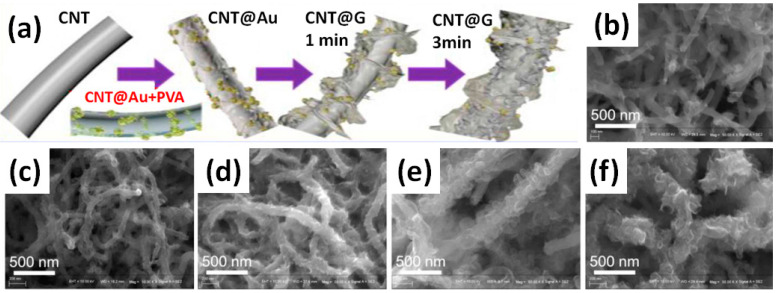
Schematic diagram of formation of CNT@Au composite with Au catalyst (**a**) and its SEM images after growth periods of about 3 min (**b**), 5 min (**c**), 6 min (**d**), 7 min (**e**) and 10 min (**f**) (Reproduced with permission of [46]. Copyright Elsevier, 2019).

**Figure 19:**
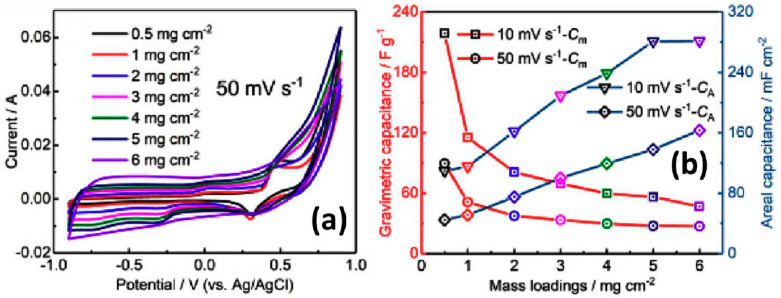
CV curves of CNT@G with different mass loadings at 50 mV/s (**a**) and calculated capacitance of the CNT@G electrode on Ni foam as a function of mass loading (**b**) (Reproduced with permission of [46]. Copyright Elsevier, 2019).

Very interesting results were obtained by Li et al. for electrodes made on the core-shell structure of G grown on CNT preliminarily covered by Au nanoparticles as catalyst (CNT@Au) [46]. Figure 18 shows a schematic diagram and obtained structures at various stages during the formation of CNT@Au composite as a function of the deposition time. Elemental Au originating from the catalyst nanoparticles was also detected by XPS [46]. CNT@G powder with graphene growth time of 5 min being pressed into Ni foam exhibited the largest CV with redox peaks and, correspondingly, the highest specific capacitance in comparison to other CNT@G. However, the reported value of the capacitance was strongly dependent on at least two factors such as the mass load and the width of the used potential window. Figure 19a illustrates that the integral area became significantly larger, but the redox peaks associated with Au catalyst became inconspicuous with increasing mass loading. Based on CV measured from −1 V to +1 V presented in Figure 19a the gravimetric (C_m_) and areal (C_a_) capacitance values at different scanning rates for CNT@G electrodes with different mass loadings were deduced (see Figure 19b). The highest gravimetric (or specific) capacitance of 218 F/g was obtained for the electrode with the lowest G@CNT mass loading of 0.5 mg/cm^2^ at 10 mV/s, but the highest areal capacitance of 281 mF/cm^2^ was obtained for the highest studied mass loading of 5–6 mg/cm^2^ also at 10 mV/s.

**Figure 20:**
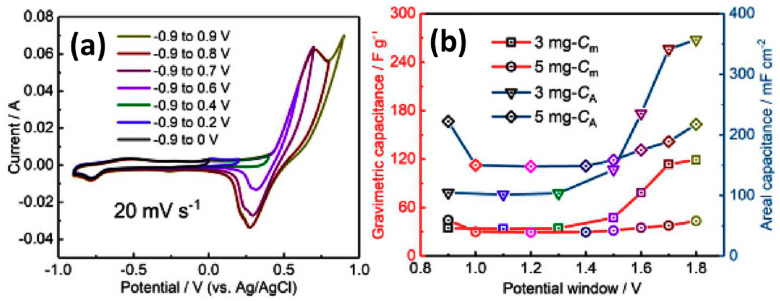
CV curves at different potential windows measured for CNT@G electrodes with 3 mg/cm^2^ mass loading at 20 mV/s (**a**) and relationship between the potential window and gravimetric (or specific) capacitance (C_m_) and areal capacitance (C_a_) for CNT@G electrodes with mass loading of 3 mg/cm^2^ and 5 mg/cm^2^ (**b**) (Reproduced with permission of [46]. Copyright Elsevier, 2019).

Regarding the potential window effect, Li et al. studied it on CNT@G electrodes with mass loadings of 3 mg/cm^2^ and 5 mg/cm^2^. A CV curve example at a scanning rate of 20 mV/s for 3 mg/cm^2^ mass loading can be seen in Figure 20a. CV curves of both electrodes show a pair of redox peaks in the negative potential range, and an additional pair of redox peaks appears with an increasing potential window on the positive side. These peaks are related to the trace amount of Au catalyst distributed on the graphene sheets. In addition, both kinds of the specific capacitance increased with the potential window width up to the maximum of 1.8 V corresponding to the range of −0.9 to 0.9 V (see Figure 20b).

**Figure 21:**
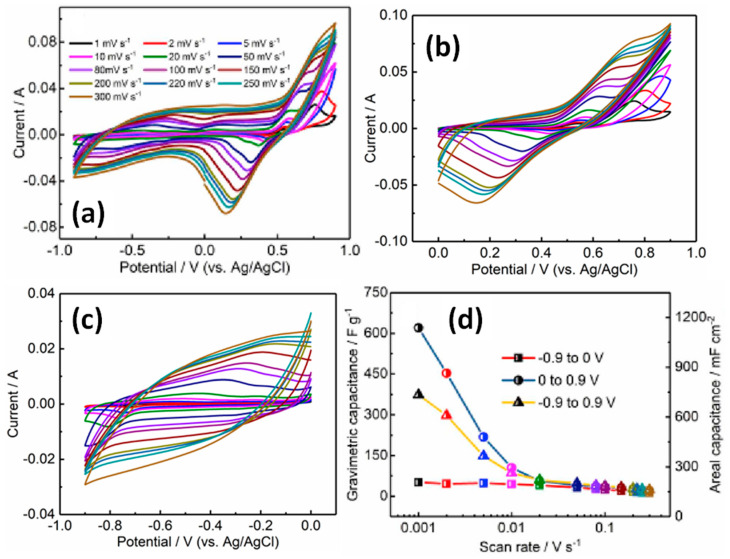
CV curves obtained at different scanning rates for CNT@G with mass loading of 3 mg/cm^2^ measured in potential window from −0.9 V to +0.9 V (**a**), from 0 to +0.9 V (**b**), from 0 to −0.9 V (**c**), and corresponding specific capacitances (**d**) (Reproduced with permission of [46]. Copyright Elsevier, 2019).

At the same time, the detailed study of CV curves recorded in the widest potential window covering the range of −0.9 V to +0.9 V (see Figure 21a), covered two smaller windows such as between 0 and +0.9 V (see Figure 21b) and between −0.9 V and 0 V (see Figure 21c) with completely different forms of CV. The negative potential window electrode works obviously as EDLC (see Figure 21c) opposite to the positive range with a visible Faradaic response (see Figure 21b). There is also a correlation with the calculated values of the specific capacitance for CNT@G/Ni electrode with mass loading of 3 mg/cm^2^. This value reaches only 51.3 F/g at 1 mV/s for EDLC in a potential window of −0.9 V ÷ 0 V, achieving very high 620 F/g for battery-like behaviour in the range 0 V ÷ +0.9 V, and the middle value of 373 F/g for EDLC with a Faradaic impact in the widest potential window −0.9 V ÷ +0.9 V (see Figure 21d). In addition, the estimated *b* parameter was different for each of all three diapasons and can be presented as 0.5 < *b*_(0 ÷ +0.9 V)_ < *b*_(−0.9 V÷ +0.9 V)_ < *b*_(−0.9 V ÷ 0)_ ~1. Moreover, as can be seen in Figure 21d, the faradaic impact to specific capacitance seen at a low scan rate disappeared with the rate increase. Furthermore, at a high scan rate >0.02 mV/s the value of the specific capacitance in all three measured potential windows becomes the same and does not exceed the lowest capacity of 51.3 F/g (see Figure 21d) [46].

#### 4.3.2. MnO_2_ Induced Pseudocapacitance

MnO_2_ is widely used for energy storage because of its high theoretical pseudocapacitance, wide potential range, and low toxicity and cost (natural abundance). The fact that MnO_2_-based composites are widely applied with neutral aqueous electrolytes being well correlated with the current environmental requirements of “green electrolytes” in supercapacitors is also important. Indeed, all reported composite electrodes based on G/rGO-CNT with MnO_2_ were tested in the Na_2_SO_4_ electrolyte as seen in Table S6 in Supplementary Materials. Comparing the values for the composites prepared with and without MnO_2_ (also shown in Table S6) it can be seen that MnO_2_ as a redox oxide can significantly increase the specific capacitance of rGO/CNT composite [47,48,49,50,51].

The highest enhancement was reported by Bi et al. for the layered structure of graphene and CNT decorated by MnO_2_ on Cu foil [47]. The long and complicated preparation of layered G/CNT with MnO_2_ structure included CVD, immersion, a thermal decomposition process, etc. However, it resulted in the specific capacitance increase from 42 F/g to 365 F/g at 1 A/g before and after MnO_2_ deposition, respectively [47].

A more popular and simple method is the use of KMnO_4_ to obtain MnO_2_ during the processing. In this way Ramezani et al. obtained the capacitance of 367 F/g at 20 mV/s for the composite of hydrazine reduced rGO, CNT, MnO_2_, graphite powder and PVDF covering graphite paper and it was twice higher than 150 F/g mentioned in the same work for rGO-CNT without MnO_2_ [48]. Electrodes on Ni foam with rGO, CNT, MnO_2_, AB, PTFE were fabricated by Liu et al. [49] and Deng et al. [51]. However, Liu et al. reported the increase from 35 F/g at 5 mV/s for rGO-CNT-AB-PTFE to 133 F/g for rGO-CNT-MnO_2_-AB-PTFE [49], using GO aerogel reduced at 800 °C. At the same time, Deng et al. used hydrazine-reduced GO, CNT, MnO_2_ AB, PTFE and reported specific capacitance of 91 F/g and 126 F/g at 0.25 A/g for electrodes without and with MnO_2_ [51]. Using urea for GO reduction and poly(1,5-diaminoanthraquinone) (PDAA) for functionalization of MnO_2_-CNT Lei et al. obtained 80 F/g and 193 F/g at 0.2 A/g for rGO, CNT, PDAA, CB, PTFE and rGO, CNT, MnO_2_, PDAA, CB, PTFE composites, respectively [50]. Preparation of rGO-CNT-MnO_2_ by the HT method at 150 °C was used by Li et al. for the fabrication of rGO, CNT, MnO_2_, AB, PTFE composite electrode on Ni foam with the final specific capacitance 336 F/g at 0.5 A/g [52]. As can be seen, all the aforementioned composite electrodes with MnO_2_ used additives and binders [48,49,50,51,52] otherwise resulting in fabrication difficulties [47]. However, Cheng et al. were able to prepare rGO-CNT-MnO_2_ freestanding electrode by simple filtration [53]. Although for that electrode, GO was reduced by hydrazine and ammonium solutions, the specific capacitance equal to 372 F/g at 10 mV/s was measured [53].

**Figure 22:**
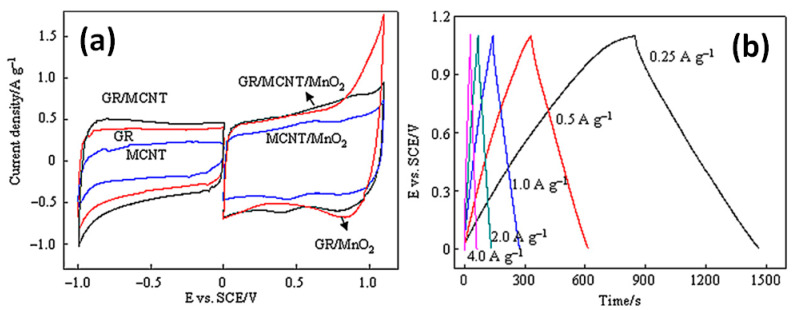
CV curves of electrodes made of rGO (mentioned as GR), on multiwall CNT (here as MCNT), their combination GR/MCNT (a, left), and their composite with MnO_2_ (GR/MCNT/MnO_2_) as well as GR/MnO_2_ and MCNT/MnO_2_ at a scan rate of 5 mV/s (**a**). GCD curves of GR/MCNT/MnO_2_ at different current densities (**b**) (Reproduced with permission of [51]. Copyright Elsevier, 2012).

It needs to be stressed here that CV and GCD curves of reported electrodes with MnO_2_ presented shapes typical for materials with dominant EDLC energy storage behaviour as can be seen in Figure 22 for G/rGO-CNT-MnO_2_-AB-PTFE reported by Deng et al. [51]. There are no significant redox peaks appearing in CV curves even at a high scan rate (see Figure 22a) and no plateau in GCD curves for these electrodes (see Figure 22b) [51]. Moreover, the *b* parameter for all aforementioned G/rGO-CNT-based composites with MnO_2_ was estimated by us to be ~0.8 that also corresponds to dominant capacitive type of storage in these electrode materials.

#### 4.3.3. Effect of Other Metal Oxides

**Figure 23:**
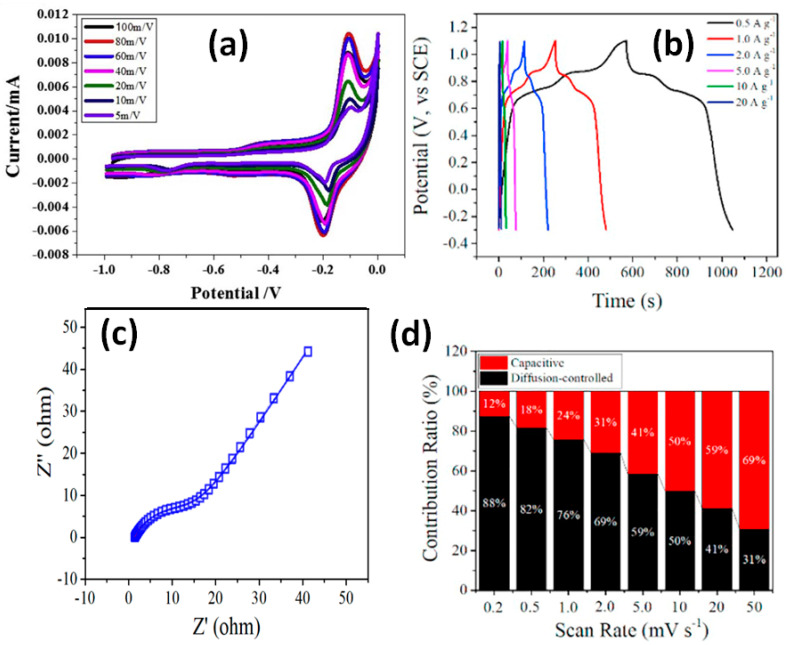
CV curves obtained at different scanning rates for rGO-CNT-Co_3_O_4_-SnO_2_-AB-PTFE composite on Ni foam (**a**) (Reproduced with permission of [54]. Copyright Elsevier, 2017). Electrochemical performance of LiMn_2_O_4_-CNT-graphene nanocomposite: GCD at different current densities (**b**), EIS Nyquist plots of the electrode after cycling (**c**). Contribution ratios of capacitive and diffusion-controlled processes at various scan rates (**d**) (Reproduced with permission of [56]. Copyright Elsevier, 2019).

In addition to MnO_2_, the influence of other metal oxides on G/rGO-CNT-based composites has been also reported [54,55,56] and summarized in Table S7 in the Supplementary Materials. Ramesh et al. mixed CNT, ammonium reduced GO, and cellulose fibres simultaneously with Co_3_O_4_ and SnO_2_, added AB, PTFE and covered Ni foam with it [54]. CV curves of such electrodes studied in KOH electrolyte presented strong redox peaks at −0.1 V ÷ −0.2 V, which, as well as GCD curve shape, cannot be attributed to EDLC (see Figure 23a) but correlated well with the faradaic impact from Co_3_O_4_ and SnO_2_ [54]. The reported specific capacitance of 215 F/g at 0.2 A/g was obtained for electrodes studied in a negative potential window from 0 to −1.0 V.

A similar diffusion-dominated energy-storage mechanism can be observed in CV curves reported by Trian et al. for electrodes on Ni foam made of rGO-CNT with Fe_2_O_3_ and mixed with CB and PTFE and supported by GCD curves (see Figure 23b) [55] or by Chen et al. for rGO-CNT with LiMn_2_O_4_ and mixed with AB, PTFE [56]. In all the cases, strong redox peaks in CV from metal oxides indicated the significant impact from faradaic materials as can be seen in Figure 23a for LiMn_2_O_4_ [56]. Moreover, GCD curves (see Figure 23b) as well as the EIS Nyquist plot with the slop close to 45° (see Figure 23c) was reminiscent the battery-type electrodes. In addition Chen et al. calculated the parameter *b* = 0.689 that is closer to *b* = 0.5 characteristic for battery-type energy storage, especially at low scan rate that can be seen in Figure 23d [56].

#### 4.3.4. G/rGO-CNT with Ni(OH)_2_

**Figure 24:**
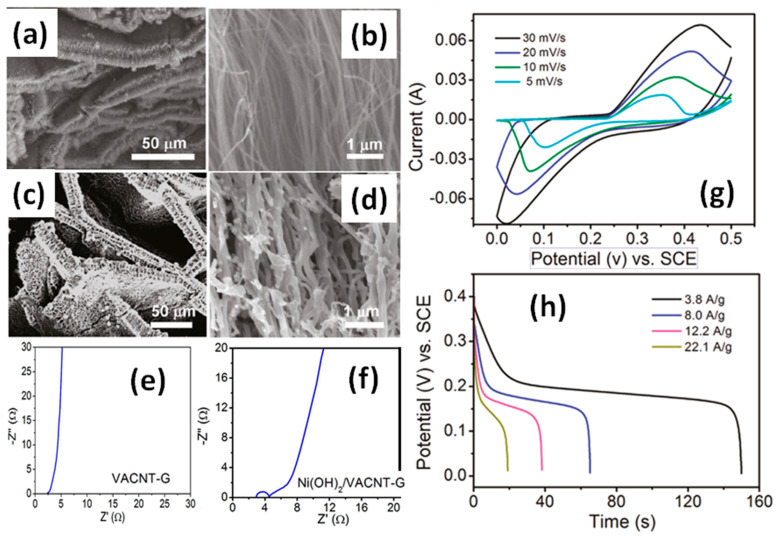
Typical SEM images of 3D pillared vertically aligned CNT (VACNT)-graphene architectures without (**a**,**b**) and with Ni(OH)_2_ (**c**,**d**). Electrochemical impedance spectra (EIS) Nyquist plots of VACNT-graphene electrode before (**e**) and after (**f**) modification by Ni(OH)_2_. CV at different scan rates (**g**) and GCD curves at various discharge current densities (**h**) (Reproduced with permission of [59]. Copyright American Chemical Society, 2011).

Nickel hydroxide is an attractive material for supercapacitor applications because of its high theoretical specific capacitance, well-defined redox behaviour and low cost. The available data for rGO-CNT before and after modification by Ni(OH)_2_ are presented in Table S8 in Supplementary Materials. The reported specific capacitance of composites with Ni(OH)_2_ has significantly higher values in comparison with other electrodes described above. Moreover, all the reported electrodes based on G/rGO-CNT with Ni(OH)_2_ were tested in KOH electrolyte, showing rather close capacitance values independent of G/rGO processing temperatures. According to Fan et al., simple mixing of Ni(NO)_3_·6H_2_O with urea and with rGO-CNT aerogel reduced at 800 °C resulted in a stable electrode on Ni foam with specific capacitance of 1208 F/g at 1 A/g, although a Ni-free rGO-CNT electrode prepared in the same way showed only 149 F/g at 1 A/g [57]. A similar value of 1320 F/g at 6 A/g was reported by Chen et al. for composite electrodes made of slurry including AB, PTFE and rGO-CNT-Ni(OH)_2_ obtained in autoclave at 120 °C [58]. A more complicated method was used by Du et al. for the preparation of vertically aligned CNT (VACNT) structure from highly ordered pyrolytic graphite at 1200 °C and G growing by pyrolysis of iron phthalocyanine (FePc) at 1000 °C (see Figure 24a) with the following Ni(OH)_2_ coating by electrochemical deposition (see Figure 24b). Specific capacitance of 110 F/g at 10 mV/s for G on a CNT structure and 1384 F/g at 5 mV/s for G on CNT and covered by Ni(OH)_2_ was measured [59].

However, all these electrodes with Ni(OH)_2_ presented CV and CGD curves very far from those for typical pseudocapacitive and particularly for EDLC materials. As presented in Figure 24c, CV curves with strong redox peaks increasing and shifting with the scan rate, typical for all G/rGO-CNT electrodes with Ni(OH)_2_, were reported by Du et al. Moreover, such electrodes have shown clearly visible plateaus typical for battery electrodes (see Figure 24d). A significant impact of faradaic contribution to G/rGO-CNT electrodes with Ni(OH)_2_ is easy to detect in EIS Nyquist plots of the G-CNT electrode before (see Figure 24e) and after (see Figure 24f) Ni(OH)_2_ deposition [59]. The slope changes from almost 90° for G-CNT with pure EDLC behaviour to almost 45° was associated with the battery. In addition, *b* parameters estimated by us are close to 0.6 that means the diffusion controlled mechanism (typical for battery-type electrodes) as the dominant one in these composites. Based on that and according to requirements from many research papers (i.e., references [14,15], etc.), other units and calculations associated with batteries (i.e., mAh instead F) need to be used for the characterization of such electrode materials. Moreover, comparison of these high specific capacitance values for such hybrid materials as well as other their parameters with that of really capacitive materials are incorrect and speculative.

## 5. Specific Energy and Power of Supercapacitors with Electrodes Based on G/rGO-CNT and Their Cycling Stability

Energy density (in Wh/cm^3^) and power density (in W/cm^3^) are known to be among the main characteristic parameters of SC for their commercial application. Therefore, the goal of research is to achieve high energy density at high power density, although in the case of the electrodes based on G/rGO-CNT these values are rarely presented, being always substituted by specific energy and power. SC specific energy (*E* in Wh/kg) and specific power (*P* in W/kg) can be calculated by using the following expressions:

(2) $$E=\frac{1}{2\times 3.6}C_{total}\Delta V^{2}\;\mathrm{or}\;E=\frac{1}{8\times 3.6}C_{single\;el.}\Delta V^{2},$$

(3) $$P=\frac{E}{\Delta t},$$

where *C_total_* and *C_single el._* are the measured capacitance of full SC and that of single electrode, respectively, ∆*V* is the operating voltage window, Δ*t* is the discharge time in hours. Thus, although the values of capacitance are very important for the SC performance, the electrolyte voltage window plays also a major role for the enhancement of specific energy as well as specific power.

However, in the case of devices with strong redox peaks in CV and plateaus in GCD curves (e.g., references [46,55,56,57]), the calculation of the specific energy cannot be done using Equations (2) and (3) valid only for capacitive materials characterized by rectangular CV and triangular GCD. That is mainly because of the non-triangular shaped GCD that is used for the calculation of energy. Whereas the specific power and energy calculation in capacitive materials is based on the area under the triangular GCD during charge discharge time, the actual energy in the battery-type materials is the area under the curved lines with plateaus. Thus, it appears that the charging energy is larger than the discharging one, reflecting the electrode reaction being not fully reversible. Hence only a portion of the energy used during the charging period was released during the discharging period. In this case, the energy efficiency considered as the ratio of discharging energy to charging energy is far smaller than 1, in contrast to that for capacitive energy storage.

**Figure 25:**
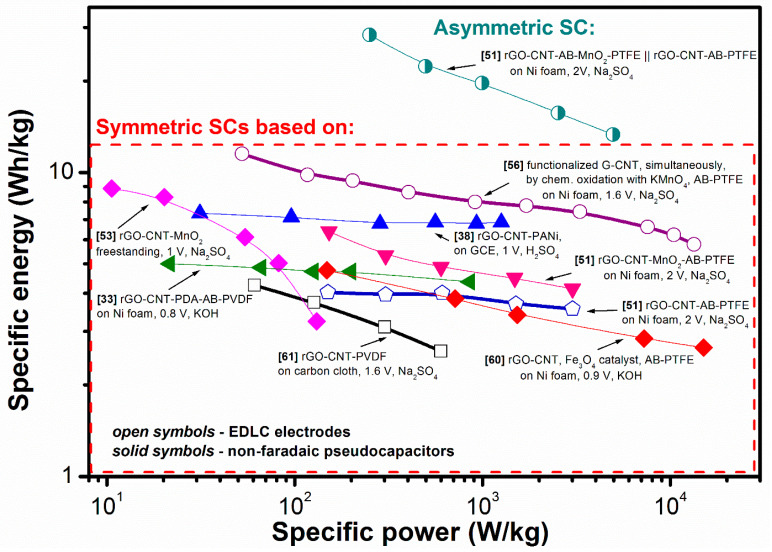
Ragone plots for reported energy storage devices using G/rGO-CNT-based electrodes preliminary tested in three electrode system.

Thus, the Ragone plot shown in Figure 25 presents only the available data of several symmetric supercapacitors made of the capacitive materials (EDLC and pseudocapacitors) and one asymmetric device that used rGO-CNT-AB-PTFE (EDLC electrode) and rGO-CNT-MnO_2_-AB-PTFE (pseudocapacitive electrode). As a result, the highest value was calculated for the asymmetric supercapacitor supporting the importance of the enlarged voltage window according to Equations (2) and (3). However, if we consider only symmetric SC with capacitive electrodes (open circle in Figure 25), a significant performance is evident to be achieved by Ding et al. for EDLC without addition of faradaic materials [21]. It is a surprise that a network of functionalized graphene nanosheets and CNT (fG/CNT) was synthesized by chemical oxidation of KMnO_4_ simultaneously with CNT showing after low temperature treatment specific energy of 11.7 Wh/kg. This value of energy density is higher than that for devices using faradaic materials, i.e., MnO_2_ [51,53], Fe_3_O_4_ catalyst [60], PANI [38], PDA [33], and it is significantly higher than that for two other SCs with EDLC electrodes [51,61]. Moreover, specific capacitance of 202 F/g at 0.5 A/g that was not too high was reported for these single fG/CNT electrodes in three-electrode configuration and the widest voltage window was not used for the electrochemical test. Thus, if Ding et al. did not find in the analysed composite the traces of MnO_2_ [21], for the preparation of which KMnO_4_ is usually used [49,50,52] and with which such an improvement could be associated, this is the best result for the G/rGO-CNT-based electrodes reported to date.

Another important characteristic for the practical application of the electrodes/full SC is their stability after charging/discharging for a long time. The cycling stability can be seen from Table 2 to be rather independent of the type of electrolyte or electrode substrate for all the reported devices, although several of them reported some fluctuations during the measurements [21,60]. While most of the values are close to 100%, there are also relatively low values of 75% and 80.5% reported for rGO-CNT with MnO_2_, AB and PTFE [51] and rGO-CNT with PANI [38], respectively. On the other hand, the cycle stability measurement of energy storage devices has to begin only after stable operation has been demonstrated and the abnormal results reported sometimes are not from the stabile cycling but rather from conditioning. In this case, electrode stabilization must be performed before the cycle stability measurements can be properly made.

## 6. Conclusions and Perspective of G/rGO-CNT-Based Composite Electrodes

The high-quality monolayer of graphene shows great potential for different applications such as miniaturized and precise micro/nano electronics, while chemically or/and thermally reduced graphene oxide provides a practical route towards lower-cost production of different rGO-based devices, particularly supercapacitors. Because GO is easily dissolved in a variety of solvents and due to high solubility of CNT achieved in GO solution, the combination of G/rGO and CNT is widely studied in SC as mixed or layered electrode materials. An amount of around 10 wt.% of CNT is generally sufficient to obtain the maximum value of the specific capacitance in case of the two-component rGO-CNT electrodes. Therefore, both freestanding and substrate supported electrodes can show high specific capacitance but only under certain/individual conditions such as the method of fabrication, type of substrate, reduction temperature or chemicals, ratio between components of the electrodes, etc. Thus, the addition of faradaic materials always increases the capacitance. However, although the compatibility of G/rGO-CNT with different polymers, metals, etc. opens up a route for a wide practical realization of functional composite materials for SC fabricated using commercial, mainly slurry-based battery technology, determining the mechanism of energy storage for each individual electrode immediately before the manufacture of the final device is essential. At the same time, talking not about electrodes only but about supercapacitors in general, high specific energy and power are crucial parameters for commercialization as well as capacitance retention. Thus, until now the highest energy and power densities were associated with hybrid devices. Based on the information described above, combining G/rGO-CNT with different additives and binders, a number of perspective composite electrodes both of capacitive and battery type can be achieved. Therefore, particular requirements to the performance of electric storage devices determine the need for detailed understanding of the relationship between the fabrication, structure and final properties of composite electrodes. In turn, the understanding of the importance of the results achieved through their comparison can be greatly facilitated if the result presentation is unified/standardized. In addition, it is obvious that the future of supercapacitors is in the asymmetric configuration, because the symmetric one has more limited behaviour that does not correspond to future needs.

## Figures and Tables

**Table 1:** Comparison of theoretical (theor.) and/or experimental (exp.) values of specific surface area (SSA), electrical conductivity (σ) and specific capacitance (C_sp_) of different carbonaceous materials such as activated carbon, graphene and single-wall carbon nanotubes (SWCNT).

	Activated Carbon	Graphene	SWCNT
**SSA, m^2^/g**	~3000 (theor. [3])	∼2600 (theor. [5])	1315 (theor. [5])
**σ, S/m**	4.6 × 10^−6^ (exp. [4])	~10^8^ (theor. [6])	~10^6^ (exp. [8])
		~10^3^ (exp. [6])	
**C_sp_, F/g**	200 (exp. [3])	~550 (theor. [7])	180 (exp. [9])

**Table 2:** Values of the initial specific capacitance (C_in_) of G/rGO-CNT-based single electrodes measured in three-electrode configuration before long-term test and capacitance retention after long cycling (ordered with number of cycles increase) reported for G/rGO-CNT-based single electrodes as well as for full devices using them.

Electrode Materials	Sub-Strate	Processing Details	Electrolyte, Voltage Window	C_in_ of Single Electrode at Current Density or Scan Rate	Number of Cycles	Capacitance Retention at Current Density or Scan Rate		Ref.
						Single Electrode	Full Cell	
rGO, CNT,	-	VF, H_6_N_2_O, NH_4_OH, KMnO_4_	Na_2_SO_4_	275 F/g	1000	95%	-	[53]
+MnO_2_			1 V	50 mV/s		50 mV/s		
rGO, CNT,	C-cloth	GO freeze-drying, 180 °C	Na_2_SO_4_	129 F/g	1000	-	90%	[61]
+PVDF		80%rGO + 10%CNT + 10%PVDF	1.6 V	0.1 A/g			0.1 A/g	
rGO, CNT,	GCE	H_6_N_2_O, 95 °C	H_2_SO_4_	359 F/g	2000	80.5%	-	[38]
+PANI		in-situ polymerization	1 V	1 A/g		50 mV/s		
rGO, CNT,	Ni foam	H_6_N_2_O, 95 °C, KMnO_4_	Na_2_SO_4_	120 F/g	2500	-	75%	[51]
+MnO_2_, AB, PTFE			2 V	1 A/g			1 A/g	
rGO, CNT,	Ni foam	HT, 180 °C, coating	KOH	165 F/g	10,000	-	98.9%	[33]
+PDA, AB, PTFE		80%AM + 10%PVDF + 10%AB	0.8 V	1 A/g			1 A/g	
CNT/G balls,	Ni foam	Fe_3_O_4_ on G by aerosolization	KOH	-	10,000	-	107.7%	[60]
+Fe_3_O_4_, PVDF		CNT by CVD, 700 °C	0.9 V				3.25 A/g	
rGO, CNT,	Ni foam	chemical oxidation, 200 °C	Na_2_SO_4_	202 F/g	20,000	103%	102%	[21]
+CB, PTFE			1.6 V	0.5 A/g		200 mV/s	200 mV/s	

## Data Availability

No new data were created or analyzed in this study. Data sharing is not applicable to this article.

## Supplementary Materials

The following are available online at https://www.mdpi.com/article/10.3390/nano11051240/s1, Table S1: Published details of capacitive G/rGO-CNT-based single electrodes measured in three-electrode configuration, Table S2: Published details of N-doped G/rGO-CNT single electrodes measured in three-electrode configuration, Table S3: Published details of single G/rGO-CNT electrodes modified by PPy measured in three-electrode configuration, Table S4: Published details of G/rGO-CNT single electrodes modified by PANI measured in three-electrode configuration, Table S5: Published details of G/rGO-CNT-based single electrodes prepared with metal catalysts measured in three-electrode configuration, Table S6: Published details of G/rGO-CNT-based single electrodes modified by MnO_2_ measured in three-electrode configuration, Table S7: Published details of G/rGO-CNT-based single electrodes modified by other metal oxides measured in three-electrode configuration, Table S8: Published details of G/rGO-CNT-based single electrodes modified by Ni(OH)_2_ measured in three-electrode configuration.

## Glossary

AB	acetylene black
AC	active carbon
AM	active material
CB	carbon black
CNT	carbon nanotubes
CV	cyclic voltammogram
CVD	chemical vapor deposition
EDLC	electric double-layer capacitors
EMI-TFSI	1-ethyl-3-methylimidazolium bis(trifluoromethylsulfonyl)imide
fG	functionalized graphene nanosheets
G	graphene
GCD	galvanostatic charge and discharge
GCE	glassy carbon electrode
GO	graphene oxide
HOPG	highly ordered pyrolytic graphite
LS	lignosulfonate
MC	mesoporous carbon
MWCNT	multiwall carbon nanotubes
NGC	Nitrogen-doped reduced graphene oxide with carbon nanotubes
PANI	polyaniline
PC	propylene carbonate
PDA	polydopamine
PDAA	poly(1,5-diaminoanthraquinone)
PNGC	porous nitrogen-doped reduced graphene oxide with carbon nanotubes
PPy	polypyrrole
PSS	poly(sodium 4-sterene sulfonate)
PTFE	polytetrafluoroethylene
PVDF	polyvinylidene fluoride
rGO	reduced graphene oxide
SC	supercapacitors
SEM	scanning electron microscopy
SSA	specific surface area
SSCNT	super short carbon nanotubes
SWCNT	single-wall carbon nanotubes
TEABF_4_	tetraethylammonium tetrafluoroborate
TEM	transmission electron microscopy
VA	vertically aligned
VGNS	vertical graphene nanosheets
VF	vacuum filtration
XPS	X-ray photoelectron spectroscopy
