# Supplementary material for: Graphene/Reduced Graphene Oxide-Carbon Nanotubes Composite Electrodes: From Capacitive to Battery-Type Behaviour
Source: Nanomaterials (Basel). 2021 May 8;11(5):1240. doi: 10.3390/nano11051240 (PMC8151991; doi:10.3390/nano11051240)
Supplement: Supplementary file 1 [file nanomaterials-11-01240-s001.zip › nanomaterials-1211787-supplementary.pdf]

**Supplementary Materials**

# Graphene/Reduced Graphene Oxide-Carbon Nanotubes Composite Electrodes: from Capacitive to Battery-type Behaviour

**Olena Okhay [1,*] and Alexander Tkach [2]**

[1] TEMA-Center for Mechanical Technology and Automation, Department of Mechanical Engineering, University of Aveiro, 3810-193 Aveiro, Portugal
[2] CICECO-Aveiro Institute of Materials, Department of Materials and Ceramic Engineering, University of Aveiro, 3810-193 Aveiro, Portugal; atkach@ua.pt
* Correspondence: olena@ua.pt

## Content

| | Page |
|---|---|
| Table S1. Published details of capacitive G/rGO-CNT-based single electrodes measured in three-electrode configuration | 2 |
| Table S2. Published details of N-doped G/rGO-CNT single electrodes measured in three-electrode configuration | 3 |
| Table S3. Published details of single G/rGO-CNT electrodes modified by PPy measured in three-electrode configuration | 3 |
| Table S4. Published details of G/rGO-CNT single electrodes modified by PANI measured in three-electrode configuration | 3 |
| Table S5. Published details of G/rGO-CNT-based single electrodes prepared with metal catalysts measured in three-electrode configuration | 4 |
| Table S6. Published details of G/rGO-CNT-based single electrodes modified by $MnO_2$ measured in three-electrode configuration | 4 |
| Table S7. Published details of G/rGO-CNT-based single electrodes modified by other metal oxides measured in three-electrode configuration | 4 |
| Table S8. Published details of G/rGO-CNT-based single electrodes modified by $Ni(OH)_2$ measured in three-electrode configuration | 5 |
| Glossary | 5 |
| List of References | 6 |

**Table S1**. Published details of capacitive G/rGO-CNT single electrodes measured in three-electrode configuration.

| Electrode materials | Sub-strate | Processing details | Electrolyte | Capacitance | Ref. |
|---|---|---|---|---|---|
| rGO,CNT,PVDF | C-cloth | 80%rGO+10%CNT+10%PVDF,180 °C | $Na_2SO_4$ | 129 F/g, 0.1 A/g | [1] |
| rGO,CNT | FS | VF, hydrozine-gas | KOH | 265 F/g, 0.1 A/g | [2] |
| rGO,CNT,PDAA,CB,PTFE | Ni foam | Urea, $KMnO_4$, 80 °C | $Na_2SO_4$ | 80 F/g, 0.2 A/g | [3] |
| rGO,CNT,AB,PTFE | Ni foam | $H_6N_2O$, 95°C, $KMnO_4$ | $Na_2SO_4$ | 91 F/g, 0.25 A/g | [4] |
| rGO,CNT | Ni foam | dried, 250 °C, pressed | KOH | 200 F/g, 0.25 A/g | [5] |
| rGO,CNT | GCE | urea | KOH | 10 F/g, 0.5 A/g | [6] |
| rGO,CNT | Ni foam | VF, dried, 250 °C, pressed | $Na_2SO_4$ | 151 F/g, 0.5 A/g | [7] |
| rGO,CNT,CB,PTFE | Ni foam | chem. oxidation, 200 °C | $Na_2SO_4$ | 202 F/g, 0.5 A/g | [8] |
| rGO,CNT,MC,Naf | C-paper | HT, 180 °C, aerogel<br>80%AM+15%MC+5% Naf | $H_2SO_4$ | 335 F/g, 0.5 A/g | [9] |
| G,CNT | Cu foil | CNT-G | $Na_2SO_4$ | 42 F/g, 1 A/g | [10] |
| rGO,CNT | GCE | $H_6N_2O$, 95 °C, in-situ polymerization | $H_2SO_4$ | 53 F/g, 1 A/g | [11] |
| rGO,CNT | fiber | vit. C, 80 °C, inject | $Na_2SO_4$ | 60 F/g, 1 A/g | [12] |
| rGO,CNT | st. steel | aerogel, 800 °C, urea, 70 °C | KOH | 149 F/g, 1 A/g | [13] |
| rGO,CNT,pp-AC | FS | 600 °C, VF<br>10%rGO+10%CNT+80%pp-AC | KOH | 214 F/g, 1 A/g | [14] |
| rGO,CNT,SDBS,PVA,AB,PTFE | Ni foam | $H_6N_2O$, coating<br>85%AM+10%AB+5%PTFE | KOH | 375 F/g, 1 A/g | [15] |
| rGO,CNT | Ni foam | VF, 300 °C, pressed | KOH | 194 F/g, 2 A/g | [16] |
| rGO,CNT | ITO | LbL, into PEI-rGO/CNT-COOH, 150°C | $H_2SO_4$ | 120 F/g, 1 mV/s | [17] |
| rGO,CNT,AB,PTFE | Ni foam | 400 °C, 800 °C, $KMnO_4$ | $Na_2SO_4$ | 35 F/g, 5 mV/s | [18] |
| rGO,CNT | Gr-paper | MW, 150°C, EPD | KCl | 87 F/g, 5 mV/s | [19] |
| rGO,CNT | Ti foil | 200 °C | $Na_2SO_4$ | 272 F/g, 5 mV/s | [20] |
| CNT,Gr | Ni foil | VA CNT on/from HOPG | KOH | 110 F/g, 10 mV/s | [21] |
| GO,CNT, Gr.powder, PVDF | Gr.-plates | $H_6N_2O$<br>80%AM+10%Gr.powder+10%PVDF | $Na_2SO_4$ | 150 F/g, 20 mV/s | [22] |
| rGO,CNT | ITO | LbL, transfer | $H_2SO_4$ | 157 F/g, 50 mV/s | [23] |
| rGO,CNT | Si | $H_6N_2O$ | KOH | 194 F/g, 50 mV/s | [24] |
| rGO,CNT,AB,PVDF | Ti plate | 100 °C, $H_6N_2O$<br>40%rGO+40%CNT+10%AB+10%PVDF | $H_2SO_4$ | 250 F/g, 50 mV/s | [25] |
| rGO,CNT | GCE | 110 °C | KOH | 132 F/g, 100 mV/s | [26] |
| rGO,CNT | FS | VF, $H_6N_2O$, $NH_4OH$ | KOH | 294 F/cm$^3$, 5 mV/s | [27] |
| rGO,CNT | fiber | vit. C, 90 °C | LiCl | 10.8 F/cm$^3$, 10 mV/s | [28] |
| rGO,CNT | fiber | vit. C, 200 °C | $H_2SO_4$ -PVA | 36.7 F/cm$^3$, 1 A/cm$^3$ | [29] |

**Table S2.** Published details of N-doped G/rGO-CNT single electrodes measured in three-electrode configuration.

| Electrode materials | Substrate | Processing details | Electrolyte | Capacitance after (before) N doping | Ref. |
|---|---|---|---|---|---|
| rGO,CNT,PDA,AB,PTFE | Ni foam | HT, 180 °C, coating 80%AM+10%PVDF+10%AB | KOH | 176 (-) F/g, 0.5 A/g | [30] |
| rGO,CNT,LS | GCE | urea, LS, 800 °C | KOH | 246 (10) F/g, 0.5 A/g | [6] |

**Table S3**. Published details of single G/rGO-CNT electrodes modified by PPy measured in three-electrode configuration.

| Electrode materials | Sub-strate | Processing details | Electrolyte | Capacitance with (without) PPy | Ref. |
|---|---|---|---|---|---|
| rGO,CNT,PPy | fiber | vit. C, PPy, twisted into springs, coated by | LiCl | 25.9 (10.8) F/cm$^3$, 0.01 V/s | [28] |
| rGO,CNT,PPy | FS | VF, $H_6N_2O$, $(NH_4)_2S_2O_8$, 95 °C | KCl | 211 (-) F/g, 0.2 A/g | [31] |
| rGO,CNT,PPy,PTFE | Gr.-sub | $H_6N_2O$ 80%AM+15%CB+5%PTFE | KCl | 361 (-) F/g, 0.2 A/g | [32] |
| G,CNT,PPy | FS | el.-chem. polymerization 0.05%G+0.05%CNT+99.9%PPy | $H_2SO_4$ | 453 (-) F/g, 5 mV/s | [33] |

**Table S4**. Published details of G/rGO-CNT single electrodes modified by PANI measured in three-electrode configuration.

| Electrode materials | Substrate | Processing details | Electrolyte | Capacitance with (without) PANI | Ref. |
|---|---|---|---|---|---|
| rGO,CNT,PANI | FS | VF, 200 °C | $H_2SO_4$ | 257 (-) F/g, 0.2 A/g | [34] |
| rGO,PANI,CNT | FS | VF, 250 °C | $H_2SO_4$ | 638 (-) F/g, 0.5 A/g | [35] |
| rGO,CNT,PANI,MC,Naf | *C*-paper | HT, 180 °C, aerogel. 80%AM+15%MC+5% Naf | $H_2SO_4$ | 987 (335) F/g, 0.5 A/g | [9] |
| rGO,CNT,PANI | GCE | $H_6N_2O$, 95 °C | $H_2SO_4$ | 359 (53) F/g, 1 A/g | [11] |
| rGO,CNT,PANI,CB,PTFE | Ni foam | $H_6N_2O$, 100 °C 75%AM+20%CB+5%PTFE | KOH | 1035 (-) F/g, 1 mV/s | [36] |
| rGO,CNT,PANI | fiber | vit. C, 200 °C, PANI onto fiber by cycling voltammetry process | $H_2SO_4$-PVA | 193 (36.7) F/cm$^3$, 1 A/cm$^3$ | [29] |

**Table S5**. Published details of G/rGO-CNT-based single electrodes prepared with metal catalysts measured in three-electrode configuration.

| Electrode materials | Sub-strate | Processing details | Electrolyte | Capacitance in F/g | Ref. |
|---|---|---|---|---|---|
| G,CNT,Co,Mo | Gr-paper | VA G by plasma-assisted method from CNT by CVD with Co/Mo | $Na_2SO_4$ | 278, 10 mV/s | [37] |
| rGO,CNT,Co,Fe/$Al_2O_3$,CB,PTFE | Ni foam | GO with $Co(NO_3)_2 \times 6H_2O$ and urea, CNT by CVD with Fe/$Al_2O_3$, 750°C 75%AM+20%CB+5%PTFE | KOH | 385, 10 mV/s | [38] |
| G,CNT,Au | Ni foam | Au on CNT, core-shell G on CNT by powder pressed | KOH-CMC | 373, 1 mV/s | [39] |

**Table S6**. Published details of G/rGO-CNT-based single electrodes modified by $MnO_2$ measured in three-electrode configuration.

| Electrode materials | Sub-strate | Processing details | Electrolyte | Capacitance with (without) $MnO_2$ | Ref. |
|---|---|---|---|---|---|
| rGO,CNT,$MnO_2$,PDAA,CB,PTF | Ni foam | Urea, $KMnO_4$, 80 °C | $Na_2SO_4$ | 193 (80) F/g, 0.2 A/g | [3] |
| rGO,CNT,$MnO_2$,AB,PTFE | Ni foam | $H_6N_2O$, 95°C, $KMnO_4$ | $Na_2SO_4$ | 126 (91) F/g, 0.25 A/g | [4] |
| rGO,CNT,$MnO_2$,AB,PTFE | Ni foam | HT,150 °C, $KMnO_4$ | $Na_2SO_4$ | 336 (-) F/g, 0.5 A/g | [40] |
| rGO,CNT,$MnO_2$,AB,PTFE | Ni foam | 400 °C, 800 °C, $KMnO_4$ | $Na_2SO_4$ | 133 (35) F/g, 5 mV/s | [18] |
| rGO,CNT,$MnO_2$ | FS | VF, $H_6N_2O$, $NH_4OH$, $KMnO_4$ | $Na_2SO_4$ | 372 (-) F/g, 10 mV/s | [41] |
| rGO,CNT,$MnO_2$,Gr.powder,PV | Gr. paper | $H_6N_2O$, 95°C, $KMnO_4$ | $Na_2SO_4$ | 367 (150) F/g, 20 mV/s | [22] |
| G,CNT,$MnO_2$ | Cu foil | LbL, CVD, 1000 °C thermal decomposition, 410 °C | $Na_2SO_4$ | 365 (42) F/g, 1 A/g | [10] |

**Table S7**. Published details of G/rGO-CNT-based single electrodes modified by other metal oxides measured in three-electrode configuration.

| Electrode materials | Substrate | Processing details | Electrolyte | Capacitance, F/g | Ref. |
|---|---|---|---|---|---|
| rGO,CNT,Cel.,$Co_3O_4$,$SnO_2$,AB,PTFE | Ni foam | $NH_4OH$, HCl, $CoCl_2 \times 6H_2O$, $SnCl_2 \times 2H_2O$, urea,150 °C, coating 70%AM+20%AB+10%PTFE | KOH | 215, 0.2 A/g | [42] |
| rGO,CNT,$Fe_2O_3$,CB,PTFE | Ni foam | 75%AM+20%CB+5%PTFE | KOH | 657, 1 A/g | [43] |
| G,CNT,$LiMn_2O_4$,AB,PTFE | Ni foam | 85%AM+10%AB+5%PTFE | $Li_2SO_4$ | 676, 0.2 mV/s | [44] |

**Table S8**. Published details of G/rGO-CNT-based single electrodes modified by $Ni(OH)_2$ measured in three-electrode configuration.

| Electrode materials | Sub-strate | Processing details | Electrolyte | Capacitance with (without) $Ni(OH)_2$ | Ref. |
|---|---|---|---|---|---|
| rGO,CNT,$Ni(OH)_2$ | st. steel | GO-CNT aerogel, 800 °C $Ni(NO)_3$×$6H_2O$, urea, 70 °C, pressed | KOH | 1208 (149) F/g, 1 A/g | [13] |
| rGO,CNT,$Ni(OH)_2$,AB,PTFE | Ni foam | GO,CNT, urea, $NiCl_2$×$6H_2O$, autoclave at 120 °C | KOH | 1320 (-) F/g, 6 A/g | [45] |
| G,CNT,$Ni(OH)_2$ | Ni foam | VA CNT on/from HOPG, 1200 °C, G by pyrolysis of FePc, 1000 °C, $Ni(OH)_2$ coating by ECD | KOH | 1384 (110) F/g, 5 mV/s | [21] |

## Glossary

| | |
|---|---|
| FS | freestanding |
| G | graphene |
| GCE | glassy carbon electrode |
| Gr | graphite |
| GO | graphene oxide |
| ITO | indium tin oxide |
| HCl | hydrochloric acid |
| HOPG | highly ordered pyrolytic graphite |
| HT | hydrothermal method |
| $H_6N_2O$ | hydrazine hydrate or hydrazine solution |
| $H_2SO_4$ | sulfuric acid |
| $HNO_3$ | nitric acid |
| $KMnO_4$ | potassium permanganate |
| KOH | potassium hydroxide |
| KCl | potassium chloride |
| LbL | layer-by-layer |
| LiCl | lithium chloride |
| $LiClO_4$ | lithium perchlorate |
| $LiMn_2O_4$ | lithium mangan dioxide |
| $Li_2SO_4$ | lithium sulfate |
| LS | lignosulfonate |
| MC | mesoporous carbon |
| AB | acetylene black |
| AC | active carbon |
| $Al_2O_3$ | aluminium oxide |
| AM | active material |
| Au | gold |
| C-paper | carbon paper |
| CB | carbon black |
| C-cloth | carbon cloth |
| Cell. | ethyl cellulose |
| chem. oxidation | chemical oxidation |
| CMC | carboxymethyl cellulose |
| CNT | carbon nanotubes |
| CNT-COOH | carboxylic acid functionalized CNT by concentrated $H_2SO_4/HNO_3$ |
| Co | cobalt |
| $Co_3O_4$ | cobalt oxide |
| $CoCl_2$×$6H_2O$ | cobalt chloride hexahydrate |
| $Co(NO_3)_2$×$6H_2O$ | cobaltous nitrate hexahydrate |
| Cu | copper |
| CVD | chemical vapor deposition |
| ECD | electrochemical deposition |
| EPD | electrophoretic deposition |
| Fe | iron |
| FePc | iron phthalocyanines |

| | |
|---|---|
| *pp*-AC | porous AC fabricated by pyrolysis, pre-carbonization, and alkali activation of biomass of pomelo peels |
| PPy | polypyrrole |
| PTFE | polytetrafluoroethylene |
| PVA | polyvinyl alcohol |
| PVDF | polyvinylidene fluoride |
| rGO | reduced graphene oxide |
| Si | silicon |
| $SnO_2$ | tin oxide |
| $SnCl_2 \times 2H_2O$ | |
| st. steel | stainless steel |
| Ti | titanium |
| VA | vertically aligned |
| VF | vacuum filtration |
| vit. C | vitamin C |
| $MnO_2$ | manganese dioxide |
| Mo | |
| MW | microwave |
| $Na_2SO_4$ | sodium sulfate |
| Naf | Nafion |
| $(NH_4)_2S_2O_8$ | ammonium persulfate |
| $NH_4OH$ | ammonia hydroxide or ammonia solution |
| Ni | nickel |
| $NiCl_2 \times 6H_2O$ | nickel chloride hexahydrate |
| $Ni(NO)_3 \times 6H_2O$ | nickel nitrate hexahydrate |
| $Ni(OH)_2$ | nickel hydroxide |
| PANI | polyaniline |
| PDA | polydopamine |
| PDAA | poly(1,5-diaminoanthraquinone) |
| PEI-rGO | reduced GO and poly(ethyleneimine) solution after removing by centrifugation of excess polymer and hydrazine hydrate |

**References**


1. Okhay, O.; Tkach, A.; Gallo, M.J.H.; Otero-Irurueta, G.; Mikhalev, S.; Staiti, P.; Lufrano, F. Energy storage of supercapacitor electrodes on carbon cloth enhanced by graphene oxide aerogel reducing conditions. *J. Energy Storage* **2020**, *32*, 101839. http://dx.doi.org/10.1016/j.est.2020.101839.
2. Lu, X.; Dou, H.; Gao, B.; Yuan, C.; Yang, S.; Hao, L.; Shen, L.; Zhang, X. A flexible graphene/multiwalled carbon nanotube film as a high performance electrode material for supercapacitors. *Electrochim. Acta* **2011**, *56*, 5115–5121. http://dx.doi.org/10.1016/j.electacta.2011.03.066.
3. Lei, Z.; Shi, S.; Lu, L. Incorporation of $MnO_2$-coated carbon nanotubes between graphene sheets as supercapacitor electrode. *ACS Appl. Mater. Interfaces* **2012**, *4*, 1058–1064. http://dx.doi.org/10.1021/am2016848.
4. Deng, L.; Hao, Z.; Wang, J.; Zhu, G.; Kang, L.; Liu, Z.-H.; Yang, Z.; Wang, Z. Preparation and capacitance of graphene/multiwall carbon nanotubes/$MnO_2$ hybrid material for high-performance asymmetrical electrochemical capacitor. *Electrochim. Acta* **2013**, *89*, 191–198. http://dx.doi.org/10.1016/j.electacta.2012.10.106.
5. Kumar, A.; Kumar, N.; Sharma, Y.; Leu, J.; Tseng, T.Y. Synthesis of free-standing flexible rGO/MWCNT films for symmetric supercapacitor application. *Nanoscale Res. Lett.* **2019**, *14*, 266. http://dx.doi.org/10.1186/s11671-019-3100-1.
6. Lin, T.T.; Lai, W.H.; Lü, Q.F.; Yu, Y. Porous nitrogen-doped graphene/carbon nanotubes composite with an enhanced supercapacitor performance. *Electrochim. Acta* **2015**, *178*, 517–524. http://dx.doi.org/10.1016/j.electacta.2015.08.048.
7. Huang, Z.D.; Zhang, B.; Liang, R.; Zheng, Q.B.; Oh, S.W.; Lin, X.Y.; Yousefi, N.; Kim, J.K. Effects of reduction process and carbon nanotube content on the supercapacitive performance of flexible graphene oxide papers. *Carbon* **2012**, *50*, 4239–4251. http://dx.doi.org/10.1016/j.carbon.2012.05.006.
8. Ding, B.; Guo, D.; Wang, Y.; Wu, X.; Fan, Z. Functionalized graphene nanosheets decorated on carbon nanotubes networks for high performance supercapacitors. *J. Power Sources* **2018**, *398*, 113–119. http://dx.doi.org/10.1016/j.jpowsour.2018.07.063.
9. Tran, V.C.; Nguyen, V.H.; Nguyen, T.T.; Lee, J.H.; Huynh, D.C.; Shim, J.J. Polyaniline and multi-walled carbon nanotube-intercalated graphene aerogel and its electrochemical properties. *Synth. Met.* **2016**, *215*, 150–157. http://dx.doi.org/10.1016/j.synthmet.2016.02.017.
10. Bi, T.; Fang, H.; Jiang, J.; He, X.X.; Zhen, X.; Yang, H.; Wei, Z.; Jia, Z. Enhance supercapacitive performance of $MnO_2$/3D carbon nanotubes graphene as a binder-free electrode. *J. Alloys Compd.* **2019**, *787*, 759–766. http://dx.doi.org/10.1016/j.jallcom.2019.02.117.

11. Huang, Y.; Zhou, J.; Gao, N.; Yin, Z.; Zhou, H.; Yang, X.; Kuang, Y. Synthesis of 3D reduced graphene oxide/unzipped carbon nanotubes/polyaniline composite for high-performance supercapacitors. *Electrochim. Acta* **2018**, *269*, 649–656. http://dx.doi.org/10.1016/j.electacta.2018.03.071.
12. Jiang, N.; Huang, F.; Xia, W.; Wei, J.; Zhou, L.; Huo, Z.; Pang, Q. Facile fabrication of rGO/CNT hybrid fibers for high performance flexible supercapacitors. *J. Mater. Sci.: Mater. Electron.* **2017**, *28*, 12147–12157. http://dx.doi.org/10.1007/s10854-017-7029-9.
13. Fan, W.; Shi, Y.; Gao, W.; Sun, Z.; Liu, T. Graphene-carbon nanotube aerogel with a scroll-interconnected-sheet structure as an advanced framework for a high-performance asymmetric supercapacitor electrode. *ACS Appl. Nano Mater.* **2018**, *1*, 4435–4441. http://dx.doi.org/10.1021/acsanm.8b00605.
14. Wu, K.; Fu, J.; Zhang, X.; Peng, X.; Gao, B.; Chu, P.K. Three-dimensional flexible carbon electrode for symmetrical supercapacitors. *Mater. Lett.* **2016**, *185*, 193–196. http://dx.doi.org/10.1016/j.matlet.2016.08.135.
15. Zhou, Y.; Hu, X.C.; Guo, S.; Yu, C.; Zhong, S.; Liu, X. Multi-functional graphene/carbon nanotube aerogels for its applications in supercapacitor and direct methanol fuel cell. *Electrochim. Acta* **2018**, *264*, 12–19. http://dx.doi.org/10.1016/j.electacta.2018.01.009.
16. Yang, Z.G.; Liu, N.N.; Dong, S.; Tian, F.S.; Gao, Y.P.; Hou, Z.Q. Supercapacitors based on free-standing reduced graphene oxides/carbon nanotubes hybrid films. *SN Appl. Sci.* **2019**, *1*, 47. http://dx.doi.org/10.1007/s42452-018-0059-y.
17. Yu, D.; Dai, L. Self-assembled graphene/carbon nanotube hybrid films for supercapacitors. *J. Phys. Chem. Lett.* **2010**, *1*, 467–470, http://dx.doi.org/10.1021/jz9003137.
18. Liu, Y.; He, D.; Duan, J.; Wang, Y.; Li, S. Synthesis of $MnO_2$/graphene/carbon nanotube nanostructured ternary composite for supercapacitor electrodes with high rate capability. *Mater. Chem. Phys.* **2014**, *147*, 141–146. http://dx.doi.org/10.1016/j.matchemphys.2014.04.020.
19. Lu, T.; Pan, L.; Li, H.; Nie, C.; Zhu, M.; Sun, Z. Reduced graphene oxide-carbon nanotubes composite films by electrophoretic deposition method for supercapacitors. *J. Electroanal. Chem.* **2011**, *661*, 270–273. http://dx.doi.org/10.1016/j.jelechem.2011.07.042.
20. Cui, X.; Lv, R.; Sagar, R.U.R.; Liu, C.; Zhang, Z. Reduced graphene oxide/carbon nanotube hybrid film as high performance negative electrode for supercapacitor. *Electrochim. Acta* **2015**, *169*, 342–350. http://dx.doi.org/10.1016/j.electacta.2015.04.074.
21. Du, F.; Yu, D.; Dai, L.; Ganguli, S.; Varshney, V.; Roy, A.K. Preparation of tunable 3D pillared carbon nanotube-graphene networks for high-performance capacitance. *Chem. Mater.* **2011**, *23*, 4810–4816. http://dx.doi.org/10.1021/cm2021214.
22. Ramezani, M.; Fathi, M.; Mahboubi, F. Facile synthesis of ternary $MnO_2$/graphene nanosheets/carbon nanotubes composites with high rate capability for supercapacitor applications. *Electrochim. Acta* **2015**, *174*, 345–355. http://dx.doi.org/10.1016/j.electacta.2015.05.155.
23. Byon, H.R.; Lee, S.W.; Chen, S.; Hammond, P.T.; Shao-Horn, Y. Thin films of carbon nanotubes and chemically reduced graphenes for electrochemical micro-capacitors. *Carbon* **2011**, *49*, 457–467. http://dx.doi.org/10.1016/j.carbon.2010.09.042.
24. Dong, X.; Xing, G.; Chan-Park, M.B.; Shi, W.; Xiao, N.; Wang, J.; Yan, Q.; Sum, T.C.; Huang, W.; Chen, P. The formation of a carbon nanotube–graphene oxide core–shell structure and its possible applications. *Carbon* **2011**, *49*, 5071–5078. http://dx.doi.org/10.1016/j.carbon.2011.07.025.
25. Zeng, F.; Kuang, Y.; Zhang, N.; Huang, Z.; Pan, Y.; Hou, Z.; Zhou, H.; Yan, C.; Schmidt, O.G. Multilayer super-short carbon nanotube/reduced graphene oxide architecture for enhanced supercapacitor properties. *J. Power Sources* **2014**, *247*, 396–401. http://dx.doi.org/10.1016/j.jpowsour.2013.08.122.
26. Lee, B.R.; Chang, D.W. Graphene/multi-walled carbon nanotubes hybrid materials for supercapacitors. *Clean Technol.* **2015**, *21*, 62–67. http://dx.doi.org/10.7464/ksct.2015.21.1.062.
27. Jiang, L.; Sheng, L.; Long, C.; Fan, Z. Densely packed graphene nanomesh-carbon nanotube hybrid film for ultra-high volumetric performance supercapacitors. *Nano Energy* **2015**, *11*, 471–480. http://dx.doi.org/10.1016/j.nanoen.2014.11.007.
28. Wang, S.; Liu, N.; Su, J.; Li, L.; Long, F.; Zou, Z.; Jiang, X.; Gao, Y. Highly stretchable and self-healable supercapacitor with reduced graphene oxide based fiber springs. *ACS Nano* **2017**, *11*, 2066–2074. http://dx.doi.org/10.1021/acsnano.6b08262.
29. Liu, D.; Du, P.; Wei, W.; Wang, H.; Wang, Q.; Liu, P. Skeleton/skin structured (RGO/CNTs)@PANI composite fiber electrodes with excellent mechanical and electrochemical performance for all solid-state symmetric supercapacitors. *J. Colloid. Interface Sci.* **2018**, *513*, 295–303. http://dx.doi.org/10.1016/j.jcis.2017.11.027.
30. Zeng, R.; Deng, H.; Xiao, Y.; Huang, J.; Yuan, K.; Chen, Y. Cross-linked graphene/carbon nanotube networks with polydopamine "glue" for flexible supercapacitors. *Compos. Commun.* **2018**, *10*, 73–80. http://dx.doi.org/10.1016/j.coco.2018.07.002.
31. Lu, X.; Dou, H.; Yuan, C.; Yang, S.; Hao, L.; Zhang, F.; Shen, L.; Zhang, L.; Zhang, X. Polypyrrole/carbon nanotube nanocomposite enhanced the electrochemical capacitance of flexible graphene film for supercapacitors. *J. Power Sources* **2012**, *197*, 319–324. http://dx.doi.org/10.1016/j.jpowsour.2011.08.112.
32. Lu, X.; Zhang, F.; Dou, H.; Yuan, C.; Yang, S.; Hao, L.; Shen, L.; Zhang, L.; Zhang, X. Preparation and electrochemical capacitance of hierarchical graphene/polypyrrole/carbon nanotube ternary composites. *Electrochim. Acta* **2012**, *69*, 160–166. http://dx.doi.org/10.1016/j.electacta.2012.02.107.
33. Aphale, A.; Maisuria, K.; Mahapatra, M.K.; Santiago, A.; Singh, P.; Patra, P. Hybrid electrodes by in-situ integration of graphene and carbon-nanotubes in polypyrrole for supercapacitors. *Sci. Rep.* **2015**, *5*, 14445. http://dx.doi.org/10.1038/srep14445.
34. Huang, Z.D.; Liang, R.; Zhang, B.; He, Y.B.; Kim, J.K. Evolution of flexible 3D graphene oxide/carbon nanotube/polyaniline composite papers and their supercapacitive performance. *Compos. Sci. Technol.* **2013**, *88*, 126–133. http://dx.doi.org/10.1016/j.compscitech.2013.08.038.

35. Liu, P.; Yan, J.; Gao, X.; Huang, Y.; Zhang, Y. Construction of layer-by-layer sandwiched graphene/polyaniline nanorods/carbon nanotubes heterostructures for high performance supercapacitors. *Electrochim. Acta* **2018**, *272*, 77–87. http://dx.doi.org/10.1016/j.electacta.2018.03.198.
36. Yan, J.; Wei, T.; Fan, Z.; Qian, W.; Zhang, M.; Shen, X.; Wei, F. Preparation of graphene nanosheet/carbon nanotube/polyaniline composite as electrode material for supercapacitors. *J. Power Sources* **2010**, *195*, 3041–3045. http://dx.doi.org/10.1016/j.jpowsour.2009.11.028.
37. Seo, D.H.; Yick, S.; Han, Z.J.; Fang, J.H.; Ostrikov, K. Synergistic fusion of vertical graphene nanosheets and carbon nanotubes for high-performance supercapacitor electrodes. *Chem. Sus. Chem.* **2014**, *7*, 2317–2324. http://dx.doi.org/10.1002/cssc.201402045.
38. Fan, Z.; Yan, J.; Zhi, L.; Zhang, Q.; Wei, T.; Feng, J.; Zhang, M.; Qian, W.; Wei, F. A three-dimensional carbon nanotube/graphene sandwich and its application as electrode in supercapacitors. *Adv. Mater.* **2010**, *22*, 3723–3728. http://dx.doi.org/10.1002/adma.201001029.
39. Li, K.; Li, H.; Li, M.; Li, C.; Su, L.; Qian, L.; Yang, B. Carbon-nanotube@graphene core-shell nanostructures as active material in flexible symmetrical supercapacitors. *Compos. Sci. Technol.* **2019**, *175*, 92–99. http://dx.doi.org/10.1016/j.compscitech.2019.03.013.
40. Li, W.; Xu, H.; Cui, M.; Zhao, J.; Liu, F.; Liu, T. Synthesis of sulfonated graphene/carbon nanotubes/manganese dioxide composite with high electrochemical properties. *Ionics* **2019**, *25*, 999–1006. http://dx.doi.org/10.1007/s11581-018-2767-0.
41. Cheng, Y.; Lu, S.; Zhang, H.; Varanasi, C.V.; Liu, J. Synergistic effects from graphene and carbon nanotubes enable flexible and robust electrodes for high-performance supercapacitors. *Nano Lett.* **2012**, *12*, 4206–4211. http://dx.doi.org/10.1021/nl301804c.
42. Ramesh, S.; Khandelwal, S.; Rhee, K.Y.; Hui, D. Synergistic effect of reduced graphene oxide, CNT and metal oxides on cellulose matrix for supercapacitor applications. *Comp. B: Eng.* **2018**, *138*, 45–54. http://dx.doi.org/10.1016/j.compositesb.2017.11.024.
43. Tian, Y.; Hu, X.; Wang, Y.; Li, C.; Wu, X. $Fe_2O_3$ nanoparticles decorated on graphene-carbon nanotubes conductive networks for boosting the energy density of all-solid-state asymmetric supercapacitor. *ACS Sustainable Chem. Eng.* **2019**, *7*, 9211–9219. http://dx.doi.org/10.1021/acssuschemeng.8b06857.
44. Chen, L.; Li, D.; Zheng, X.; Chen, L.; Zhang, Y.; Liang, Z.; Feng, J.; Si, P.; Lou, J.; Ci, L. Integrated nanocomposite of $LiMn_2O_4$/graphene/carbon nanotubes with pseudocapacitive properties as superior cathode for aqueous hybrid capacitors. *J. Electroanal. Chem.* **2019**, *842*, 74–81. http://dx.doi.org/10.1016/j.jelechem.2019.04.056.
45. Chen, X.; Chen, X.; Zhang, F.; Yang, Z.; Huang, S. One-pot hydrothermal synthesis of reduced graphene oxide/carbon nanotube/$\alpha$-$Ni(OH)_2$ composites for high performance electrochemical supercapacitor. *J. Power Sources* **2013**, *243*, 555–561. http://dx.doi.org/10.1016/j.jpowsour.2013.04.076.
